# Large‐scale evolution of body temperatures in land vertebrates

**DOI:** 10.1002/evl3.249

**Published:** 2021-08-12

**Authors:** Matthew O. Moreira, Yan‐Fu Qu, John J. Wiens

**Affiliations:** ^1^ Center for Environmental and Marine Studies, Department of Biology University of Aveiro Aveiro Portugal; ^2^ Department of Ecology and Evolutionary Biology University of Arizona Tucson Arizona USA; ^3^ Jiangsu Key Laboratory for Biodiversity and Biotechnology, College of Life Sciences Nanjing Normal University Nanjing Jiangsu China

**Keywords:** Body temperature, diel activity, endothermy, evolution, niche conservatism, phylogeny, physiology, vertebrate

## Abstract

Body temperature is a crucial variable in animals that affects nearly every aspect of their lives. Here we analyze for the first time largescale patterns in the evolution of body temperatures across terrestrial vertebrates (tetrapods: including amphibians, mammals, birds and other reptiles). Despite the traditional view that endotherms (birds and mammals) have higher body temperatures than ectotherms, we find they are not significantly different. However, rates of body‐temperature evolution are significantly different, with lower rates in endotherms than ectotherms, and the highest rates in amphibians. We find that body temperatures show strong phylogenetic signal and conservatism over 350 million years of evolutionary history in tetrapods, and some lineages appear to have retained similar body temperatures over time for hundreds of millions of years. Although body temperatures are often unrelated to climate in tetrapods, we find that body temperatures are significantly related to day‐night activity patterns. Specifically, body temperatures are generally higher in diurnal species than nocturnal species, both across ectotherms and, surprisingly, across endotherms also. Overall, our results suggest that body temperatures are significantly linked to phylogeny and diel‐activity patterns within and among tetrapod groups, rather than just climate and the endotherm‐ectotherm divide.

**Impact Summary**

Body temperature is a crucial variable in animals that affects nearly every aspect of their lives. For example, in ectotherms (like amphibians and many reptiles), many species are only active at certain environmental temperatures, whereas in endotherms (like birds and mammals) considerable energy is invested in maintaining relatively constant body temperatures across different environmental temperatures. Here, we analyze for the first time the evolution of body temperatures across all major groups of land vertebrates (tetrapods), including amphibians, mammals, birds, and other reptiles. We find several surprising results. First, despite the traditional view that endotherms have higher body temperatures than ectotherms, we find that body temperatures are not significantly different between these two groups of species. However, we do find that rates of evolution of body temperatures among species differ significantly between ectotherms and endotherms, with ectotherms showing more rapid evolution of different body temperatures among species than birds or mammals. The fastest rates of body‐temperature evolution occur in amphibians. We find that body temperatures generally reflect the evolutionary history of species, with more closely related species tending to share more similar body temperatures, and with some groups retaining similar body temperatures over remarkably long timescales (hundreds of millions of years). Surprisingly, we also find that body temperatures are higher in diurnal (day‐active) species than nocturnal (night‐active) species, in both ectotherms and endotherms. This pattern is unexpected because variation in body temperatures among endotherm species is generally unrelated to the different climatic conditions where they occur, implying that their body temperatures should also be unrelated to day‐night fluctuations in environmental temperatures. Overall, our results show that body temperatures in land vertebrates can be strongly related to evolutionary history and day‐night activity patterns, whereas the traditional division in body temperatures between endothermic and ectothermic vertebrates was not upheld.

Body temperature is a crucial component of life in animals. Depending on the species and conditions, body temperatures may reflect environmental temperatures, behavioral thermoregulation, and/or diverse physiological mechanisms for heating and cooling (e.g., Huey et al. 2003; Morrison and Nakamura 2019). Body temperatures are important for organismal performance, and therefore for fitness (Angilletta et al. 2002). For many species, individuals are only active under certain temperatures (which can differ strongly among species), and almost all aspects of their behavior, ecology, life history, and physiology can be sensitive to body temperature (Huey and Stevenson 1979; Angilletta et al. 2002; Angilletta 2009). Species’ activity patterns may also be restricted by their preferred body temperatures to certain microhabitats (e.g., sun vs. shade; surface vs. underground) and certain times of the day and year (e.g., mornings in summer). For endotherms such as birds and mammals, considerable energy goes into maintaining body temperatures at a relatively constant level across environmental temperatures (Bennett and Ruben 1979; Nespolo et al. 2011; Fristoe et al. 2015). Thus, body temperatures are crucial in general, and may be especially important to species’ survival as global temperatures rise, for both ectotherms and endotherms (e.g., Sinervo et al. 2010; Bonebrake et al. 2020).

In land vertebrates (tetrapods), much research has focused on the origins of endothermy in mammals and birds, not body temperatures specifically (Bennett and Ruben 1979; Ruben 1995; Hayes and Garland 1995; Bennett et al. 2000; Farmer 2000; Koteja 2000; Angilletta, Jr. and Sears 2003; Pörtner 2004; Grigg et al. 2004; Nespolo et al. 2011; Little and Seebacher 2014; Lovegrove 2017). The evolution of endothermy is associated with many changes, such as metabolic rate, stamina, and aerobic capacity (Bennett and Ruben 1979; Hayes and Garland 1995; Ruben 1995). Nevertheless, there has been important work on macroevolutionary patterns in body temperatures in some groups, such as mammals (Lovegrove 2012) and lizards (Grigg and Buckley 2013; Meiri et al. 2013). For example, these studies showed strong phylogenetic signal in body temperatures within both groups, and that body size does not significantly impact temperatures in either. However, such large‐scale evolutionary patterns have not been studied across tetrapods.

Here, we address several fundamental but unanswered questions about the large‐scale evolution of body temperatures in tetrapods. Are body temperatures significantly related to phylogeny? Based on studies within lizards and mammals (see above), we predict strong phylogenetic signal across tetrapods. What body temperatures are ancestral for tetrapods and the major groups within them? Given strong signal, ancestral tetrapod body temperatures may reflect those of groups closest to the tetrapod root (e.g., amphibians, basal lepidosaurs). Do rates of change in body temperatures vary across major tetrapod clades, such that changes in body temperature among species are more rapid in some groups than others? Endotherms are often thought to have body temperatures that are higher, more stable, and presumably less variable (among species) compared to ectothermic vertebrates (Bennett and Ruben 1979; Nespolo et al. 2011). For example, Fristoe et al. (2015) stated that birds and mammals “maintain their high, relatively constant body temperatures in the face of wide variation in environmental temperature” (p. 15,934). Therefore, we predict that endotherms have higher body temperatures than ectotherms, and lower rates of body‐temperature evolution among species. To our knowledge, these questions have not been explicitly addressed with phylogenetic methods. Finally, given that body temperatures are largely uncorrelated with large‐scale climatic temperatures in many major tetrapod groups (Qu and Wiens 2020), might diel (day‐night) activity patterns help explain this variation instead? Analyses in lizards found higher body temperatures in diurnal species than nocturnal species (Meiri et al. 2013), but it is unclear whether this dichotomy applies to other ectothermic tetrapods, or endotherms. Other ectotherms might show this pattern because of the difficulty of achieving higher body temperatures at night, whereas endotherms might if less energy is needed to maintain low body temperatures at night or high body temperatures by day (Crompton et al. 1978; Hut et al. 2012; Levesque et al. 2018). Alternatively, endotherms might show similar body temperatures among species across environmental temperatures (e.g., Morrison and Nakamura 2019), including different climates and diel‐activity patterns.

We take advantage of several new resources to address these questions. These include new compilations of data on body temperatures and diel activity for many tetrapods (Anderson and Wiens 2017; Qu and Wiens 2020), extensive time‐calibrated phylogenies within major groups (Jetz et al. 2012; Zheng and Wiens 2016), and new methods for estimating ancestral trait values and rates of trait evolution (Smaers et al. 2016; Smaers and Mongle 2017). We analyze data on body temperature, diel activity, and phylogeny for 1721 tetrapod species.

## Methods

### DATA COLLECTION

Body temperature data were assembled from literature sources (Dataset S1). For most groups, we used a recent compilation of tetrapod data (Qu and Wiens 2020), which included species with diel‐activity data and that were included in large‐scale, time‐calibrated phylogenies. We assembled here published body‐temperature data for turtles, crocodilians, and additional amphibians (methods in Appendix S1 in Supporting Information). The data presented and analyzed for each species is a mean across sampled individuals or the mean across localities for species sampled from multiple locations (or midpoints of ranges among individuals, when these were the only data available).

Body‐temperature data for ectotherms were generally from active (not sleeping) animals in the field (especially since their body temperatures can depend on environmental temperatures). However, data from a few amphibian species (*n* = 5), crocodilians (*n* = 5), and turtles (*n* = 3) were from active animals under laboratory conditions (in which they could thermoregulate). We found no significant differences between body temperatures for animals in the field and those under laboratory conditions for these groups (Appendix S1).

For endotherms, much of the data were from compilations (Clarke and Rothery 2008; Clarke et al. 2010). These compilations used published data, preferentially using studies in which measured individuals were conscious, normothermic, and resting (not actively exercising) when temperatures were taken. Thus, temperatures for ectotherms should generally be from individuals that are (potentially) behaviorally thermoregulating in the field, whereas those for endotherms should generally be for individuals that are awake but do not have elevated temperatures because they are exercising at the moment of data collection. Endotherms included several species with data from the laboratory and others with data from the field. In Appendix S2, we address the potential effects of exercise and captivity on body temperatures. We conclude that exercise impacts body temperatures in endotherms (but not ectotherms) whereas captivity does not for either group (Appendix S2). We consider the most appropriate comparisons to be between thermoregulating ectotherms and resting endotherms.

We obtained diel‐activity data on all species with body‐temperature data, primarily using a recent survey across tetrapods (Anderson and Wiens 2017). Species were classified as diurnal, nocturnal, arrhythmic, or crepuscular (Dataset S2), with definitions following Anderson and Wiens (2017). Diurnal and nocturnal species were those primarily active during the day or night, respectively. Species active both during the day and night (or with seasonal shifts in activity) were classified as arrhythmic. Species primarily active during dawn and/or twilight were considered crepuscular. For species with body‐temperature data but lacking diel‐activity data, additional sources for diel data were used (Jones et al. 2009; Meiri et al. 2013; AmphibiaWeb 2019). Within each group, most sampled species were diurnal or nocturnal (>75%; Table S1). We focused in particular on comparing species in these two categories, since it is unclear how body temperatures should be related to arrhythmic or crepuscular activity.

### PHYLOGENY

We assembled a time‐calibrated phylogeny for all sampled species. The tree combined estimates for higher‐level vertebrate phylogeny (Alfaro et al. 2009), amphibians (Pyron and Wiens 2013), mammals (Rolland et al. 2014), lepidosaurs (Zheng and Wiens 2016), turtles (Jaffe et al. 2011), crocodilians (Oaks 2011), and birds (Jetz et al. 2012). We used a large‐scale vertebrate supertree (Anderson and Wiens 2017) and replaced subtrees within groups with subtrees that included additional species with body‐temperature data. For lepidosaurs, the crown age from the subtree (277.6 million years ago; Zheng and Wiens 2016) was older than the stem age from the backbone tree (270.3 million years ago; Anderson and Wiens 2017). We adjusted the crown age from 277.6 million years ago (Zheng and Wiens 2016) to 267.2 million years ago, following a recent phylogenomic estimate (Irisarri et al. 2017).

The final tree included 1721 species (Dataset S3), with data on body temperature and diel activity for all species. We obtained matching data from 571 mammal species (101/167 families; 60.5%), 11 crocodilians (3/3 families; 100%), 474 birds (84/249; 33.7%), 30 turtles (11/14; 78.6%), 518 lepidosaurs (39/68; 57.4%), and 117 amphibians (13/74; 17.6%). For taxonomy, we used recent references for amphibians (AmphibiaWeb 2019), mammals (Burgin et al. 2018), birds (Clements et al. 2019), and crocodilians, turtles, and lepidosaurs (Uetz et al. 2019). Our species sampling was broadly proportional to the extant species richness of each group (amphibians: 8285 species; mammals: 6399; lepidosaurs: 11,053; turtles: 361; crocodilians: 26; birds: 10,721; based on sources listed above). However, mammals were somewhat overrepresented and amphibians were somewhat underrepresented in terms of body‐temperature data. These sampling biases should not generally be problematic here, since we analyzed each group separately for most tests. Our sampling was also incomplete within groups, especially for amphibians. However, we sampled most major clades in each group (except the rare, species‐poor caecilians), even if not all families were sampled. We address the general issue of incomplete taxon sampling at the end of the Methods.

We also tested the main results using an alternative tree (Dataset S4), incorporating different phylogenetic estimates within amphibians, birds, mammals, and lepidosaurs. The details of the tree and analyses are given in Appendix S3, including a justification for why we used each estimate in the primary analyses as opposed to the alternative analyses. Overall, the results were very similar to those using the primary tree (Appendix S3).

### TESTING PHYLOGENETIC SIGNAL

We tested if body temperatures significantly covaried with the phylogeny, as expected given phylogenetic niche conservatism (Wiens et al. 2010). We used the function “fitContinuous” to estimate λ (Pagel 1999) in the R package *geiger* version 2.0.6.2 (Harmon et al. 2008; Pennell et al. 2014). Traits can also be strongly conserved without showing phylogenetic signal (Revell et al. 2008), but this possibility was strongly rejected for these data by our overall results for tetrapods. Conservatism can also be supported by other lines of evidence, including continuous retention of similar trait values for long timescales based on ancestral reconstructions on time‐calibrated phylogenies (Anderson and Wiens 2017). We briefly discuss this pattern also.

### ANCESTRAL RECONSTRUCTIONS

We estimated ancestral body temperatures for each node in the tree and rates of body temperature evolution for each major clade. We performed these tasks primarily using a multiple variance Brownian motion (BM) model (Smaers et al. 2016; Smaers and Mongle 2017). This model was implemented with the Markov chain Monte Carlo (MCMC) approach in the R package *evomap* version 0.0.0.9000 (Smaers and Mongle 2019), using the function “mvBM.” This method allowed for considerable variability in rates and variances across the tree, including branch‐specific rates of evolution (Appendix S4). We used 20,000,000 generations, sampling every 64,000 generations, with a 15% burn‐in. We ran a total of 20 chains and tested for convergence using the R package *coda* version 0.19.1 (Plummer et al. 2006) and Tracer version 1.7.1 (Rambaut et al. 2018). We assumed that the MCMC chains converged when we obtained an effective sample size >200 (Drummond et al. 2007), and all results reported met this criterion. We calculated the 95% highest posterior density interval (HPDI) for reconstructions of major nodes using the function “hdi” from the R package *HDInterval* version 0.2.2 (Meredith and Kruschke 2020).

We also used alternative methods to reconstruct body‐temperature evolution, which gave similar results (detailed methods and results in Appendix S4). These were implemented in the R packages *l1ou* version 1.42 (Khabbazian et al. 2016) and *mvMORPH* version 1.1.0 (Clavel et al. 2015). The package *l1ou* estimates shifts between optima in Ornstein‐Uhlenbeck (OU) models. In contrast, *mvMORPH* allows comparison of the fit of the data to constant‐variance BM and OU models, and allows ancestral reconstructions using the best‐fitting model. We primarily focused on the *evomap* results since this approach allowed for multiple variance BM models. Furthermore, we found that BM models had better support for these data than OU models (Appendix S4).

### EFFECT OF ENDOTHERMY ON BODY TEMPERATURES

We tested for significant differences in body temperatures between endotherms (mammals, birds) and ectotherms (amphibians, lepidosaurs, crocodilians, turtles), given the hypothesis that these two endotherm clades have higher body temperatures (Bennett and Ruben 1979; Nespolo et al. 2011). We also tested for significant differences overall among major tetrapod clades (amphibians, mammals, lepidosaurs, turtles, crocodilians, birds). We used phylogenetic ANOVA (Garland et al. 1993) using the function “phylANOVA” in the R package *phytools* version 0.6.99 (Revell 2012). Species were the units of the analyses. We recognize that there is some heterogeneity in endothermy and ectothermy within these major divisions, such as pythons and sea turtles with myogenic endothermy (Hayes and Garland 1995). However, most prior literature has focused on the dichotomy between birds and mammals and the other four groups (see Introduction). We therefore tested this dichotomy here.

### DIFFERENCES IN RATES AMONG CLADES

We tested for differences in rates of body‐temperature evolution between endotherms and ectotherms among clades. We obtained overall rate estimates for body temperature for each of the six major tetrapod clades using the function “mvBM.getRate” in *evomap*. This method provides the mean value of the distribution of estimated rates, taking into account all internal and terminal branches for each clade (Appendix S4). We then assigned clades as endotherms or ectotherms, and tested for differences between these two groups of clades using phylogenetic ANOVA. For this analysis, we built a reduced tree among the six major clades (Dataset S5). We reduced the tree to one species per clade using the R package *ape* version 5.3 (Paradis and Schliep 2019). The choice of species has no impact on the results.

We also tested for differences in rates of body‐temperature evolution between endotherms and ectotherms at the species‐level (using *evomap* and the function “mvBM.getRate”). We obtained the mean of the distribution of estimated rates for each terminal branch. Species‐level rates are provided in Dataset S6. We then assigned species as being endotherms or ectotherms, and tested for differences between these two groups using phylogenetic ANOVA. In contrast to these clade‐level results, results based on phylogenetic ANOVA of species‐level rate estimates were non‐significant both with amphibians (F = 0.34, *P* = 0.976) and without (F = 0.02, *P* = 0.994). In this case, differences in rates among species within clades seemed to obscure any potentially significant patterns among clades. For example, the mean rate in mammals was strongly influenced by just six species with very fast rates (Table S2). Therefore, we focused on the clade‐level results rather than the species‐level results. Nevertheless, the mean species‐level rates for each clade were generally similar to the clade‐level rates (Table S2).

### EFFECT OF DIEL ACTIVITY ON BODY TEMPERATURES

We tested for significant differences in body temperatures between the different diel‐activity states using phylogenetic ANOVA. We first tested the effect of diel activity for all species, and primarily focused on comparing nocturnal and diurnal species (those in which we predict that body temperatures will differ). Thus, species with other states were initially excluded. We also performed the same analysis within each clade (mammals, birds, lepidosaurs, turtles, amphibians). We did not analyze crocodilians separately, since all sampled species are nocturnal. We also performed analyses separately among endothermic species (birds, mammals) and ectothermic species (all other tetrapods). We also performed supplementary analyses including all four diel‐activity states.

### EFFECTS OF INCOMPLETE TAXON SAMPLING

We note that many readers may have reasonable concerns about the limited taxon sampling relative to the large size of the clades analyzed here. Therefore, we performed a series of analyses in which we randomly sampled only 10% of the species in the tree (details in Appendix S5). However, we ensured that major clades were represented (as in the main analyses). The results (Results, Appendix S5) were generally similar to those from the main analyses. Some phylogenetic ANOVA results were non‐significant in some replicates, consistent with simulations suggesting that limited taxon sampling reduces statistical power, but rarely leads to false positives (Ackerly 2000).

## Results

Body temperatures showed very strong phylogenetic signal across tetrapods (λ = 0.98), with a value very close to the maximum possible (λ = 1.00). Phylogenetic signal was also high within most clades (λ = 0.849−0.948; Table S2). However, it was substantially lower in amphibians (λ = 0.744). The value in crocodilians was very low (λ = 0.0) but this was mostly likely an artifact of the small number of crocodilian species (*n* = 11; see Table S2). These overall results were robust using alternative trees (Table S3).

**Figure 1:**
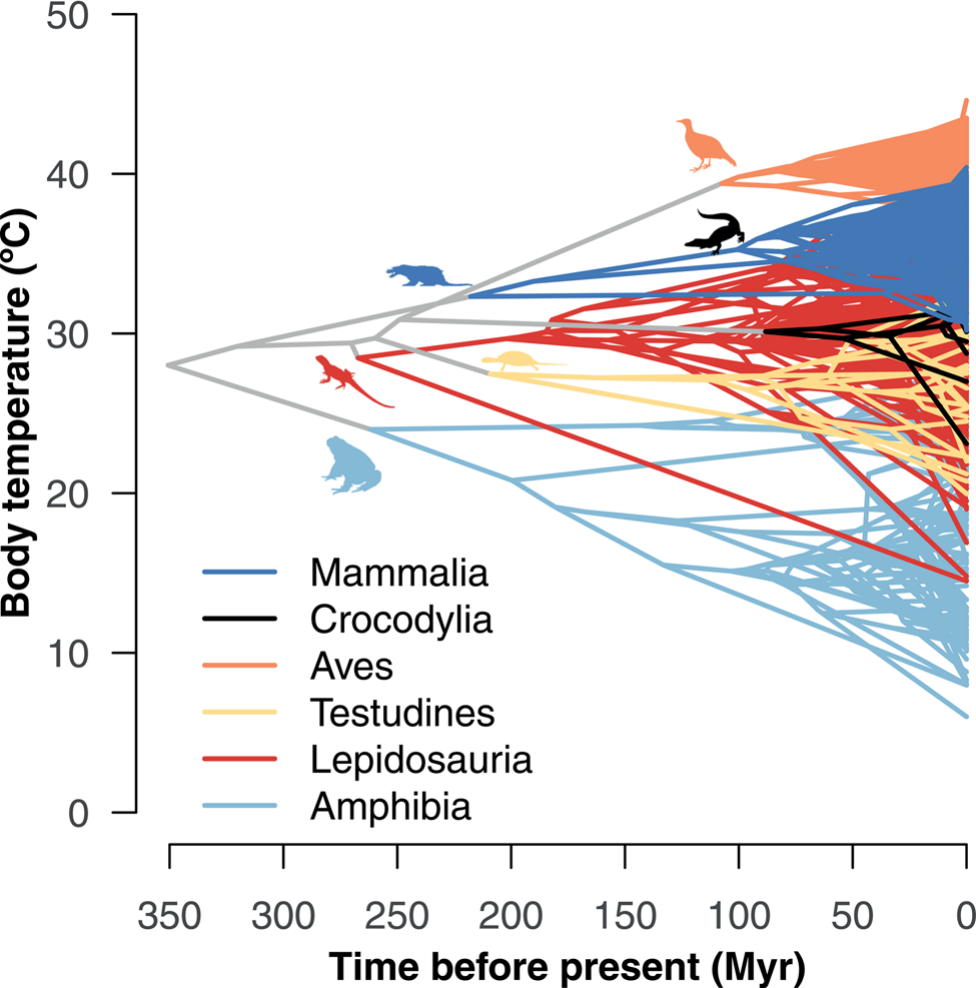
Evolution of body temperatures across tetrapod phylogeny. Reconstructions are based on the multiple variance Brownian Motion model on the primary tree (*n* = 1721). Temperatures are given in Dataset S1. Time is in millions of years before the present (Myr). Lepidosaurs include lizards, snakes, and the tuatara. Silhouettes courtesy of PhyloPic: T. Michael Keesey (mammal; Public Domain Dedication 1.0 license), B. Kimmel (crocodilian; Public Domain Dedication 1.0 license), George Edward Lodge (bird; Public Domain Dedication 1.0 license), Scott Hartman (turtle; Creative Commons Attributions 3.0 Unported license), Michael Scroggie (lepidosaur; Public Domain Dedication 1.0 license) and Steven Traver (amphibian; Public Domain Dedication 1.0 license)

We inferred ancestral body temperatures for major clades (Fig. 1) based on a multiple variance Brownian Motion model. The estimated body temperature for the crown‐node ancestor of tetrapods was 28.0°C (95% highest posterior density interval, HPDI = 23.7−32.4). The crown‐node ancestors of crocodilians (*θ* = 30.1°C [27.3−32.9]), lepidosaurs (*θ* = 28.5°C [24.0−32.9]) and turtles (*θ* = 27.5°C [23.6−31.3]) had estimated body temperatures similar to the inferred tetrapod ancestor (Fig. 1). The ancestors of mammals (*θ* = 32.3°C [28.8−35.6]) and birds (*θ* = 39.4°C [37.5−41.4]) evolved higher body temperatures, whereas the ancestor of amphibians (*θ* = 24.0°C [20.2−27.9]) evolved lower body temperatures (Fig. 1). There was then the evolution of much lower and higher temperatures within many of these groups, including much colder temperatures in some amphibians and a few lepidosaurs (e.g., tuatara) and much higher body temperatures in birds and some mammals and lepidosaurs (Fig. 1). These results were largely consistent using alternative trees (Appendix S3) and using alternative approaches for reconstructing character evolution (Appendix S4).

**Figure 2:**
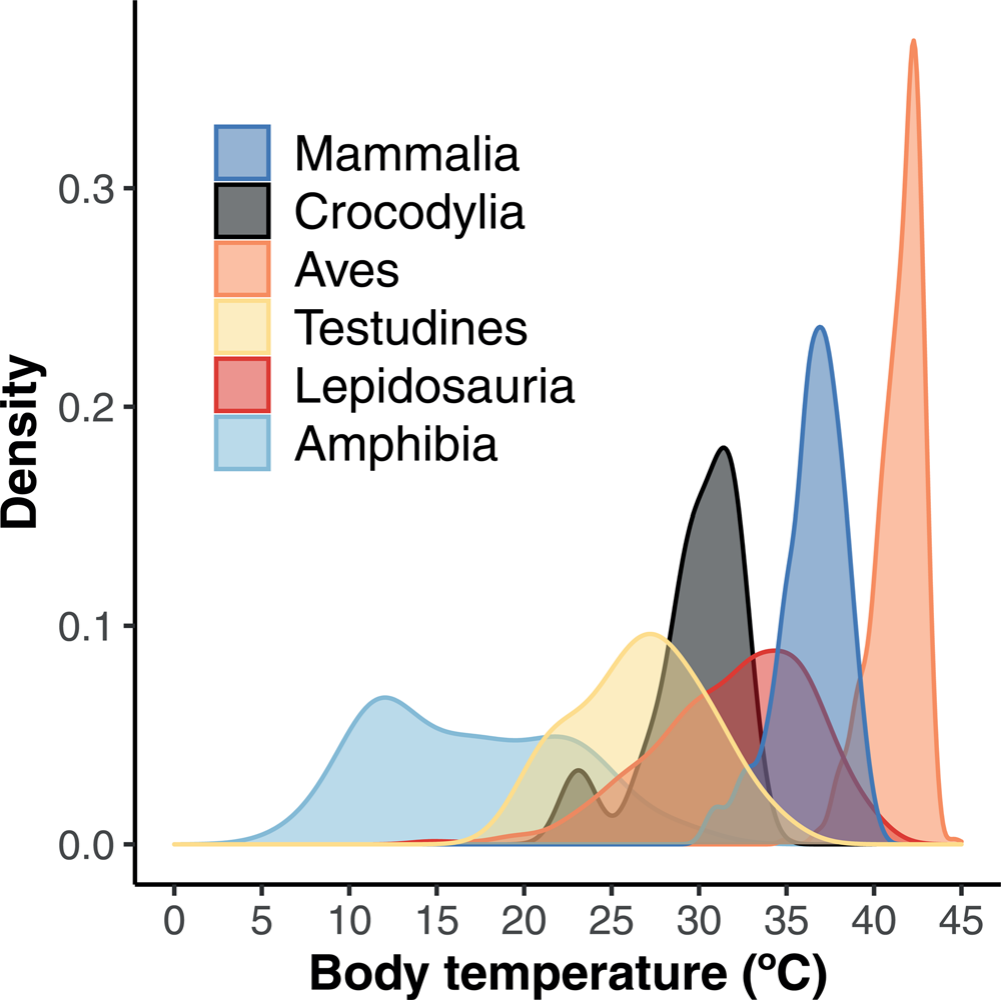
Distribution of body temperatures among major tetrapod clades. Density plots are shown for each clade, including mammals (*n* = 571), crocodilians (*n* = 11), birds (*n* = 474), turtles (*n* = 30), lepidosaurs (*n* = 518), and amphibians (*n* = 117). Data for each species are in Dataset S1. Lepidosaurs include lizards, snakes, and the tuatara

Based on phylogenetic ANOVA of data for individual species, body temperatures (Fig. 2; Dataset S1) differed significantly among the major tetrapod clades (F = 1272.55, *P* = 0.032; *n* = 1721). Surprisingly, the difference between ectotherms and endotherms was not strictly significant (F = 1421.56, *P* = 0.058). Instead, the overall differences among groups were mainly related to the high mean body temperatures of birds (*x̅* = 41.4°C; *n* = 474) and mammals (*x̅* = 36.4°C; *n* = 571) relative to low values in amphibians (*x̅* = 17.0°C; *n* = 117; Table S4).

**Figure 3:**
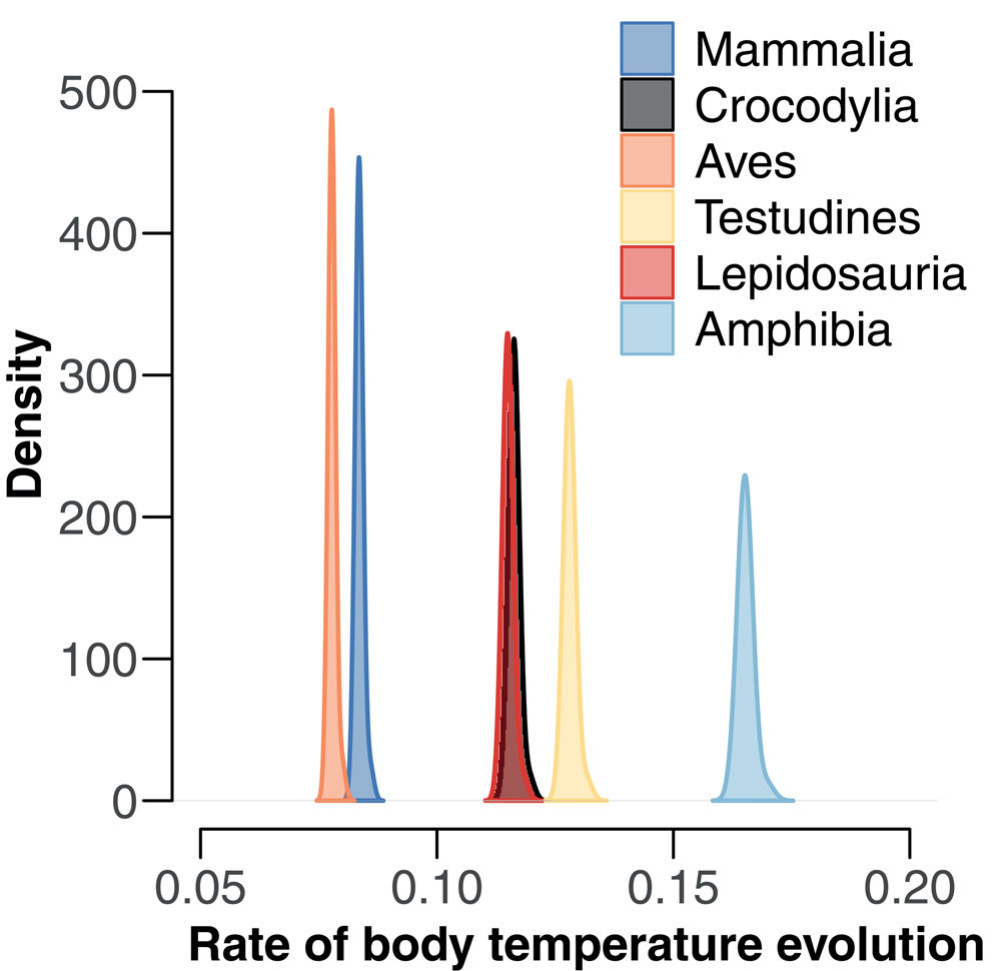
Distribution of estimated rates (density) at the clade‐level for rates of body temperature evolution (σ^2^) in the major tetrapod clades. The distribution of estimated rates is based on all internal and terminal branches for each clade. Lepidosaurs include lizards, snakes, and the tuatara

Rates of body‐temperature evolution within each clade (Fig. 3) were also estimated using the multiple‐variance Brownian‐motion model. Rates were much lower in mammals (σ^2^ = 0.084) and birds (σ^2^ = 0.078) than amphibians (σ^2^ = 0.165). Crocodilians (σ^2^ = 0.116), lepidosaurs (σ^2^ = 0.115), and turtles (σ^2^ = 0.128) showed intermediate rates, similar to the overall rate across the full tree (σ^2^ = 0.107). These results were robust to using alternative trees (Appendix S3). Based on a phylogenetic ANOVA of these clade‐level rates, rates in ectotherms and endotherms were significantly different (F = 8.16, *P* = 0.040). The difference was stronger after excluding amphibians (F = 40.51, *P* = 0.023), thus comparing only among amniote clades.

**Figure 4:**
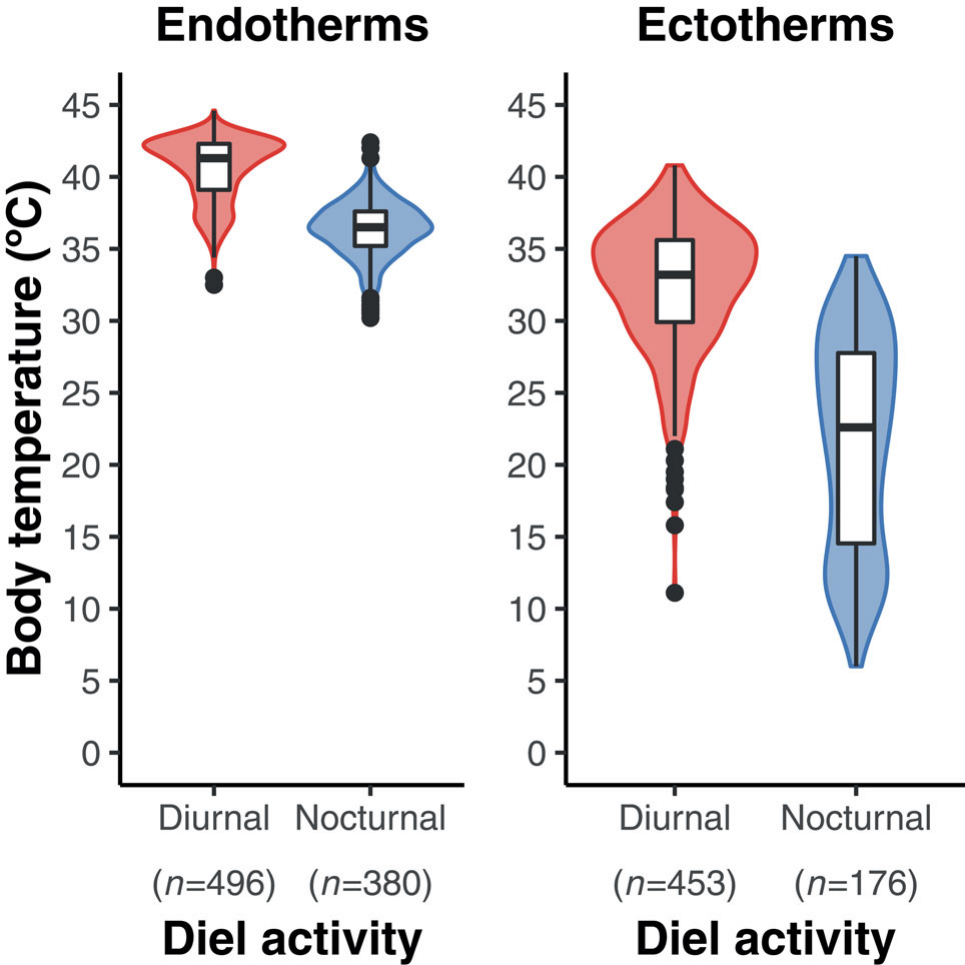
Body temperatures of endotherms and ectotherms differ between diurnal and nocturnal species. Sampled endotherms include 876 species and ectotherms include 629 species. Boxplots show the median (thick horizontal line), 25th and 75th percentiles (upper and lower edges of box), range (highest and lowest values excluding outliers; vertical lines), and outliers (black circles). Lepidosaurs include lizards, snakes, and the tuatara

Mean body temperatures among species were consistently lower for nocturnal than diurnal species across tetrapods, and in all major groups (Table 1). Nevertheless, using phylogenetic ANOVA, we did not find significant differences between body temperatures of nocturnal and diurnal species across tetrapods (F = 204.33, *P* = 0.311, *n* = 1505), nor within turtles (F = 3.64, *P* = 0.211, *n* = 23) or amphibians (F = 2.13, *P* = 0.519, *n* = 106). Note that crocodilians are all nocturnal. There were significant differences within birds (F = 45.71, *P* = 0.003, *n* = 407) and lepidosaurs (F = 98.79, *P* = 0.034, *n* = 489), and mammals approached significance (F = 38.1, *P* = 0.054, *n* = 469). Importantly, there were significant differences between nocturnal and diurnal species (Fig. 4) across ectotherms (F = 504.29, *P* = 0.004; *n* = 629) and across endotherms (F = 855.81, *P* = 0.008; *n* = 876). We also performed analyses including all four diel‐activity states (Table S5 and S6), which yielded similar results.

**Table 1:** Mean body temperatures are higher among diurnal species than nocturnal species, across tetrapods and within major groups. Crocodilians are excluded here because they are all nocturnal. Note that most (but not all) species in each group are either primarily nocturnal or primarily diurnal (Table S1). Mean temperatures for other states are given in Dataset S7. Lepidosaurs include lizards, snakes, and the tuatara

Group	Diel activity	Mean body temperature (°C)	Sample size (species)
Tetrapods	Nocturnal	31.6	556
	Diurnal	36.7	949
Amphibians	Nocturnal	16.5	98
	Diurnal	19.5	8
Mammals	Nocturnal	36.1	357
	Diurnal	37.3	112
Lepidosaurs	Nocturnal	27.6	64
	Diurnal	32.9	425
Turtles	Nocturnal	22.8	3
	Diurnal	27.2	20
Birds	Nocturnal	39.7	23
	Diurnal	41.5	384
Ectotherms	Nocturnal	21.5	176
	Diurnal	32.4	453
Endotherms	Nocturnal	36.4	380
	Diurnal	40.6	496

These analyses were performed on our primary phylogeny. We also addressed the robustness of the results to using alternative phylogenies within each of the most species‐rich tetrapod groups (i.e., amphibians, birds, lepidosaurs, mammals). Analyses using these alternative trees gave very similar results. The details of the trees and the results of these analyses are given in Appendix S3.

We also performed analyses using only 10% of the sampled species (Appendix S5). Based on 10 random selections of species, our conclusions were generally upheld, including the overall strong phylogenetic signal, the approximate ancestral values for major clades, the difference in rates between endotherms and ectotherms, and the differences in body temperatures between diurnal and nocturnal species (e.g., in endotherms and ectotherms).

## Discussion

In this study, we examined large‐scale patterns in the evolution of body temperatures in tetrapods. Our results revealed several surprising findings. We found that body temperatures show a strong phylogenetic signal despite the very deep timescale of tetrapod evolution (350 million years). Contrary to expectations, we found that endotherms do not have significantly higher body temperatures than ectotherms. However, endotherms did have significantly lower rates of body‐temperature evolution than ectotherms, especially when amphibians were excluded. Amphibians were highly divergent in having lower mean body temperatures and higher rates of body‐temperature evolution than other tetrapods. Thus, a crucial but unappreciated dichotomy in body‐temperature evolution in tetrapods is between amphibians and amniotes. We also found significant differences in mean body temperatures between nocturnal and diurnal species in many tetrapod groups (Table 1; Fig. 4). This pattern represents another important but underemphasized dichotomy in body‐temperature evolution. Intriguingly, this dichotomy occurs in both ectotherms and endotherms. We also estimated the overall patterns of body‐temperature evolution across tetrapod phylogeny. We discuss each of these patterns below and how they are interrelated.

### THE DAY‐NIGHT DIVIDE

The divergence between body temperatures of nocturnal and diurnal species across all ectothermic tetrapods has not been previously documented, but does make intuitive sense. Attaining high body temperatures at night may be difficult for ectotherms (Crompton et al. 1978). Thus the highest body temperatures among ectotherms should be confined to diurnal species (i.e. some lizards). Indeed, this diel divide in body temperatures was previously shown in lizards (Meiri et al. 2013).

What is surprising is that we found a similar divergence in body temperatures across endotherms also, and this pattern was significant within birds and approached significance in mammals. This is surprising because endotherms are widely thought to maintain similar body temperatures across different environmental conditions. For example, body temperatures in birds and mammals show relationships with climatic temperature variables (Qu and Wiens 2020) that are non‐significant (birds) or very weak (mammals; *r* ^2^ < 0.04). Birds are especially intriguing because they show a very limited range of body temperatures overall, but a highly significant difference between body temperatures in nocturnal and diurnal species. One potential explanation for this overall pattern in endotherms is that there is less energy expenditure required to maintain cooler body temperatures for nocturnal species by night and less energy to maintain hotter temperatures by day in diurnal species (Crompton et al. 1978; Hut et al. 2012; Levesque et al. 2018). Indeed within (at least some) mammal species, body temperatures may show daytime increases and nighttime decreases (Levesque et al. 2016). However, diurnality can also reduce energy expenditures in otherwise nocturnal mammals, in some species (van der Vinne et al. 2015). Thus, this simple explanation may not apply, or at least not universally. Amphibians and turtles also show the dichotomy in temperatures but not significantly, and this weaker effect might be due to limited sample sizes (turtles), high variability among species (amphibians), and/or the use of aquatic habitats that reduce diel temperature fluctuations. The day‐night divide in body temperatures might also weaken relationships between large‐scale climatic temperatures and body temperatures in all of these groups. Overall, future work should focus on explaining the causes of this dichotomy, in ectotherms but especially endotherms.

### THE ENDOTHERM‐ECTOTHERM DICHOTOMY

Our results offer a modern, phylogenetic test of the long‐standing idea that endotherms have higher body temperatures than ectotherms (e.g., Bennett and Ruben 1979; Nespolo et al. 2011; Fristoe et al. 2015). Surprisingly, this pattern was not significantly supported (*P* = 0.058), and had even less support using the alternative tree (*P* = 0.069). We found that birds have higher temperatures overall, but there was broad overlap between mammals and lepidosaurs (Fig. 2). Furthermore, some lepidosaurs with very high body temperatures (teiids, iguanians) overlapped with birds. Indeed, there were no statistically significant differences between mammals or birds and any other tetrapod group (except amphibians; Table S4). Thus, these results do not support the long‐held view that birds and mammals have significantly higher body temperatures than ectothermic tetrapods.

We did find that endotherms (birds and mammals) had lower rates of body‐temperature evolution than ectotherms. Although we have not seen this pattern explicitly predicted before, it is consistent with the idea that mammals and birds maintain relatively constant body temperatures across different environmental conditions (within and among species). A recent study (Qu and Wiens 2020) confirmed that body temperatures in birds and mammals showed no or weak relationships with large‐scale climate, depending on the variable. In contrast, lepidosaur body temperatures showed a significant relationship with the hottest annual climatic temperatures (Qu and Wiens 2020). Most importantly, amphibian body temperatures showed significant, relatively strong relationships with climatic temperatures (Qu and Wiens 2020). We note that relationships between body temperatures and climatic temperatures were not previously tested across crocodilian and turtle species: we performed analyses here for these two groups and found that neither showed significant relationships (Appendix S6).

### THE AMPHIBIAN‐AMNIOTE DICHOTOMY

In terms of body‐temperature evolution, the exceptional tetrapods are not only the endothermic birds and mammals, but also amphibians. Mean body temperatures in amphibians are lower overall than in other tetrapod groups, and evolved at a faster rate. These high rates may be explained (at least in part) by the stronger climate‐physiology relationships in amphibians relative to other groups (Qu and Wiens 2020). Amphibians may also show greater variability in their temperatures (and higher rates of evolution) because most species are thought to have relatively limited or no thermoregulation, especially salamanders (Brattstrom 1979; Olalla‐Tárraga and Rodríguez 2007; Solano et al. 2016). Furthermore, amphibians have mean body temperatures that are much lower than other tetrapods, especially in salamanders (Fig. 2). For example, some salamander and frog species breed at night during early spring at high latitudes (e.g., spring peepers; *Pseudacris crucifer*), when air temperatures are barely above freezing (AmphibiaWeb 2019). Thus, body temperatures in some amphibians are very low both because of large‐scale climate and when they are active. Yet, many amphibian species also occur in the lowland tropics (including many frogs and some salamanders). These shifts in temperature regimes may strongly contribute to the variability in body temperatures among amphibian species, and their high rates of change relative to other tetrapod groups.

### LARGE‐SCALE PHYLOGENETIC PATTERNS

We also estimated the overall patterns in the evolution of body temperatures across tetrapod phylogeny. We estimated that the ancestral body temperature of tetrapods was in the upper 20s (28.0°C; HPDI in Results), similar to the estimates for the ancestors of lepidosaurs, turtles, and crocodilians, with amphibians lower (24.0°C) and mammals higher (32.3°C), and birds much higher (39.4°C). We suggest that many of these patterns, and the overall pattern of strong phylogenetic signal, may be partly explained by diel activity. The relatively low temperatures estimated for the ancestors of most major tetrapod groups (low 20s to low 30s) are consistent with those of many living nocturnal species. Nocturnal activity is estimated to be ancestral in tetrapods, and may have been conserved from that ancestor to the present day (350 million years) in many nocturnal mammals, amphibians, crocodilians, and lepidosaurs (Anderson and Wiens 2017). Diurnal activity, associated with higher body temperatures, seems to have evolved more recently in lepidosaurs and birds and appears to have been maintained for >100 million years in each of these groups (Anderson and Wiens 2017). Overall, day‐night activity patterns are also strongly conserved across tetrapod phylogeny (Anderson and Wiens 2017), with λ > 0.95. This conservatism in diel behavior may help explain phylogenetic signal in body temperatures across tetrapod phylogeny. At the same time, conservatism in body‐temperature evolution may also help reinforce conservatism in diel‐activity patterns, especially for ectotherms (e.g., if species that are adapted to high diurnal temperatures avoid nighttime activity because of lower temperatures).

We acknowledge that some extinct taxa may have had body temperatures that were very different from those inferred here. Our results are based on inferred values for specific nodes in tetrapod phylogeny, especially the ancestors of major living clades. Our inferences are not inconsistent with the idea that some extinct taxa may have had body temperatures that were very different from these inferred ancestors and from living members of these groups (e.g., a terrestrial, diurnal crocodilian with very high body temperatures). The crucial distinction is that such exceptional fossil taxa are not necessarily the ancestors of clades living today (especially if they had derived traits that were different from these ancestors). Furthermore, we are not arguing that particular ancestors had specific values (e.g., we suggest that the most recent tetrapod ancestor likely had a value in the upper 20s, not exactly 28.0°C). Nevertheless, we inferred ancestors of many major clades that had body temperatures corresponding to a relatively narrow range of values, relative to the full range observed among extant species. We also performed a preliminary analysis that included estimated body temperatures from 14 non‐avian dinosaurs, and found little impact on our overall results.

### LONG‐TERM CONSERVATISM OF THE LOCAL‐SCALE NICHE

Finally, our results strongly support the idea that local‐scale aspects of the ecological niche can be conserved over surprisingly deep timescales. In addition to finding a strong phylogenetic signal, we find that many extant species appear to have retained body temperatures similar to those inferred for the ancestor of tetrapods, through a seemingly continuous chain of ancestors going back ∼350 million years (Fig. 1). These include many extant amphibians, crocodilians, lepidosaurs, and turtles, all with body temperatures from ∼25−30°C. Much of the early literature on niche conservatism focused on large‐scale climatic niches (Peterson et al. 1999), and the idea that these climatic niches were conserved but only over relatively short timescales (e.g. millions of years but not tens or hundreds of millions of years). Our study adds to the list of different aspects of the non‐climatic, local‐scale ecological niche that can show strong conservatism over deep timescales (∼350−900 million years), including habitat (Wiens 2015), diel activity (Anderson and Wiens 2017), and diet (Román‐Palacios et al. 2019). Body temperature may be a particularly pivotal aspect of the niche, since it may strongly influence nearly every aspect of an organisms’ biology (Huey and Stevenson 1979; Angilletta et al. 2002; Angilletta 2009).

## AUTHOR CONTRIBUTIONS

M.O.M. and J.J.W. designed research; M.O.M. and Y.F.Q. collected data; M.O.M. analyzed data; M.O.M. and J.J.W. wrote the paper.

## DATA ARCHIVING

All data are included with this submission as Supporting Information in Datasets S1–S8. These data files are also available on Dryad (https://doi.org/10.5061/dryad.76hdr7sx3).

## CONFLICT OF INTEREST

The authors declare no conflict of interest.

## Supporting information

Supplementary Material
