# Supplementary material for: Large‐scale evolution of body temperatures in land vertebrates
Source: Evol Lett. 2021 Aug 12;5(5):484–94. doi: 10.1002/evl3.249 (PMC8484719; doi:10.1002/evl3.249)
Supplement: Supplementary file 1 — Supplementary Material [file EVL3-5-484-s001.zip › supplementary_material_22July2021.docx]

***Supporting Information***

**This file includes:**

**Appendix S1.** Body temperature data for amphibians, turtles, and crocodilians.

**Appendix S2.** Effects of exercise and captivity on body temperatures

**Appendix S3.** Alternative phylogeny and results

**Appendix S4.** Alternative methods for ancestral reconstruction

**Appendix S5.** Taxon sampling analyses

**Appendix S6.** Relationships between climate and body temperature in crocodilians and turtles

**Appendix References**

**Figure S1.** Estimated rates of body-temperature evolution within each major clade using the alternative tree.

**Figure S2.** Reconstructed evolution of body temperatures using evomap on the alternative tree.

**Figure S3.** Estimated rate shifts in body temperature across tetrapods using 11ou.

**Figure S4.** Ancestral reconstructions of body temperature using mvMORPH.

**Table S1.** Percentage of sampled species with each diel activity state in each clade.

**Table S2.** Estimated phylogenetic signal and evolutionary rates for body temperature in tetrapods for the primary tree.

**Table S3.** Estimated phylogenetic signal and evolutionary rates for body temperature in tetrapods for the alternative tree.

**Table S4.** Results of phylogenetic ANOVA testing for differences in body temperatures across major tetrapod clades, using the primary tree.

**Table S5.** Results of phylogenetic ANOVA testing for a relationship between diel activity and body temperatures, using all four diel-activity states on the primary tree.

**Table S6.** Results of phylogenetic ANOVA testing for a relationship between diel activity and body temperatures, using all four diel activity states and the alternative tree.

**Table S7.** Estimated ancestral body temperatures and the 95% highest posterior density interval for the ancestral node of each major clade.

**Table S8.** Results of Ornstein-Uhlenbeck model testing.

**Table S9.** Location of rate shifts and support for each shift for the two best-fitting OU models.

**Table S10.** Relative support for different models of body-temperature evolution.

**Table S11.** Model comparisons for the evolution of body temperature (using 11ou) after excluding amphibians.

**Other supporting information for this manuscript (in separate files, not in this file) includes the following:**

**Dataset S1.** Body temperature data

**Dataset S2.** Diel activity data

**Dataset S3.** Primary phylogeny used

**Dataset S4.** Alternative phylogeny used

**Dataset S5.** Reduced version of primary phylogeny

**Dataset S6.** Species-level rates of body-temperature evolution

**Dataset S7.** Mean body temperatures for each diel activity state within major clades

**Dataset S8.** Climatic data for crocodilian and turtle species

**Dataset S9.** Resampled trees with 10% taxon sampling

**Dataset S10.** Results of subsampling analyses on the primary tree

**Appendix S1. Body temperature data for amphibians, turtles, and crocodilians.**

**Methods.** To obtain data on body temperatures for amphibians, turtles, and crocodilians, we searched the literature using both Google and Google Scholar. Data for amphibians were mostly from a recent study (Qu and Wiens 2020). However, to increase the sample size of species, we searched for body temperature data for additional species for this clade. Overall, the search was restricted to species for which we knew information on diel activity and phylogeny were available (Anderson and Wiens 2017). We searched for body temperature data for 35 amphibian species, 88 turtle species, and 19 crocodilian species. Searches were conducted from June 2019 to August 2019. For each search, we used as keywords “preferred body temperature” and the species’ binomial. The term “preferred body temperature” refers to the body temperature that individuals attempt to reach by behavioral thermoregulation (Hill et al. 2016). Whenever we did not find data for a given species using these keywords, we used “body temperatures” and the species’ binomial instead. Generally, we used body temperatures for active animals in the field (Qu and Wiens 2020). We were able to include some additional species by using temperature data taken under laboratory conditions, in which individuals were able to choose their preferred temperature (amphibians: 29 additional species with field data vs. 5 species with laboratory data; crocodilians: 6 vs. 5; turtles: 27 vs. 3). These laboratory measurements should be equivalent to those taken from active, behaviorally thermoregulating animals in the field. Nevertheless, we also performed a phylogenetic ANOVA for each clade to check for significant differences (see below). Overall, we obtained body temperature data for 34 amphibian species (added to the existing data from 83 species from a previous study; Qu and Wiens 2020), 30 turtle species, and 11 crocodilian species (Dataset S1). To obtain a single temperature value for each species, we used the mean value for body temperatures across sampled individuals (either the reported mean or the mean calculated here). If only a range of body temperatures among individuals was reported, we used the midpoint.

To test for any effects associated with using temperature data obtained in the laboratory, we tested for significant differences between species body temperatures that were collected under laboratory conditions and those in the field. We used phylogenetic ANOVA (Garland et al. 1993), with species as the units of the analyses. This approach was applied using the function “phylANOVA” in the R package *phytools* v0.6.99 (Revell 2012).

**Results.** We found no significant differences between modes of data collections (field vs lab) for amphibians (F=0.39, *P*=0.761, *n*=34), turtles (F=0.06, *P*=0.781, *n*=30), and crocodilians (F=1.11, *P*=0.194, *n*=11).

**Appendix S2. Effects of exercise and captivity on body temperatures**

Here we address whether exercise and captivity might influence body temperatures for endotherms, ectotherms, and the comparison between them. Much of the endotherm data used here were from previous compilations (Clarke and Rothery 2008; Clarke et al. 2010)­. These compilations preferentially used data in which measured individuals were conscious, normothermic, and resting (not actively exercising) when body temperatures were taken. Therefore, we addressed how use of resting (versus exercising) individuals might impact the results.

For endotherms, previous studies have found that exercise can increase body temperatures somewhat. For example, a study found that birds show a 1.5ºC increase in mean body temperatures when comparing data from species measured when resting (*x̅*=38.5ºC; *n*=203 species) to those measured during exercise (*x̅*=40.0ºC; *n*=724; Prinzinger et al. 1991). Comparisons of resting and exercising conspecifics for mammals show a 1.9ºC increase (*x̅*=36.7ºC vs. *x̅*=38.6ºC; *n*=50 species; Aschoff 1981; Prinzinger et al. 1991). By contrast, body temperatures in ectotherms are not thought to increase during exercise (Glazier 2009; Tattersall et al. 2016), especially since they generally have negligible internal heat production (Seebacher 2009; Tattersall 2016). Resting temperatures should be more appropriate for clade-level comparisons between endotherms and ectotherms to avoid the bias from increased temperatures caused by exercise itself.

We also checked if body temperatures for endotherms were comparable between field and laboratory conditions. Since the data for most endotherms in this study were compiled from two previous studies (Clarke and Rothery 2008; Clarke et al. 2010), we checked those sources to find the original literature to determine whether species had data from the field or lab. Also, we corrected the body temperature data for 12 bird species and 17 mammal species based on the original sources. When the original literature was not available using the previous approach, we used the species name and “body temperature” as keywords to search for data on the Web of Science, Bing Scholar, and Baidu Scholar. We checked the first five pages of results for each search engine. However, we were not able to find the literature sources for all species. Based on the available literature, we identified how the body temperature data were collected (i.e. from the laboratory vs field) for 148 bird species (104 from the lab vs 44 from the field) and 505 mammal species (470 vs 35; Dataset S1). Finally, we tested for significant differences between mean body temperatures between those species with data from the field and those species with data from the lab. We used phylogenetic ANOVA (Garland et al. 1993), with the function “phylANOVA” in the R package *phytools* v0.6.99 (Revell 2012). We compared birds and mammals separately, given the differences in body temperatures between these groups (see Results). Unfortunately, the sampling for mammals was strongly biased towards species with data from the lab. Therefore, any difference observed between mean field and body temperatures across all species might be related to which species had data from the field, and not biological differences caused by captivity. Given this, for mammals the phylogenetic ANOVA included only species from orders with at least two species with data from the lab and two species with data from the field. We found no significant differences for birds (F=0.08, *P*=0.853, *n*=148) or mammals (F=4.28, *P*=0.278, *n*=325). Thus, we concluded that whether data were obtained from the field or laboratory should not impact the body temperature data for endotherms. Our results above (Appendix S1) for ectotherms also suggest that there are no significant differences between field and laboratory body temperatures for these groups.

**Appendix S3. Alternative phylogeny and results**

**Methods.** To address the robustness of our results to alternative phylogenetic hypotheses, we developed an overall tetrapod tree with alternative relationships within the four largest clades (Qu and Wiens 2020). Alternative subtrees were used for amphibians (Jetz and Pyron 2018), mammals (Faurby et al. 2018), lepidosaurs (Tonini et al. 2016), and birds (Jetz et al. 2012). We used the consensus trees for amphibians and squamates previously generated (Qu and Wiens 2020). We did not know of alternative, time-calibrated, species-level phylogenies for crocodilians or turtles that were as extensive as those used in the main tree (and these two groups had relatively few sampled species regardless).

We focused on using different alternative phylogenies from entirely different studies rather than analyzing a posterior distribution of trees from a single study. We expect the summary of the phylogeny-based results based on a distribution of trees from the same analysis to be very similar to that based on the consensus of those trees. In contrast, we expect more meaningful impacts of topology and branch lengths on the results when using trees from different studies.

We acknowledge that we could have used these subtrees in the primary tree, and not as alternative trees. In general, we considered the subtree selected for the primary tree to be a better or equivalent choice. For amphibians, the primary tree used (Pyron and Wiens 2013) is more resolved than the secondary tree (Jetz and Pyron 2018), even for taxa represented by sequence data. For squamates, the primary tree (Zheng and Wiens 2016) is based on many more loci for the higher-level relationships. Furthermore, at least one family (Teiidae) that is traditionally recognized is monophyletic in the primary tree (Zheng and Wiens 2016) but is not monophyletic in the secondary tree (Tonini et al. 2016). For birds, the primary and alternative trees are from the same source (Jetz et al. 2012), but the backbone tree in the primary analysis is from a more recent study (Hackett et al. 2008) whereas the backbone phylogeny in the alternative tree is from a somewhat older study (Ericson et al. 2006). For mammals, more species were included in the subtree used in the primary tree (i.e. 12 species were missing in the alternative tree, see below). Furthermore, the higher-level relationships in the primary tree (Rolland et al. 2014) are based on a large-scale multi-locus analysis (Meredith et al. 2011), which we consider preferable. Another alternative mammalian phylogeny has recently became available (Upham et al. 2019), but this appeared to be missing some species, even relative to the secondary tree.

For most groups, all the sampled species were represented in the alternative tree.

However, 12 species were absent in the alternative mammalian tree, and these were dropped from the overall tetrapod tree (*Bos taurus*, *Bubalus bubalis*, *Capra hircus*, *Cavia porcellus*, *Chaetophractus nationi*, *Fukomys amatus*, *Urva auropunctata*, *Micaelamys namaquensis*, *Ningaui yvonneae*, *Ovis aries*, *Oxymycterus dasytrichus*, *Taurotragus oryx*). The final alternative tree included 1709 species.

The comparative methods used on the alternative tree were identical to those used in the main analyses. For example, phylogenetic signal was again tested using the function “fitContinuous” in *geiger* v2.0.6.2 (Harmon et al. 2008; Pennell et al. 2014). Ancestral reconstructions were performed using *evomap* v0.0.0.9000 (Smaers and Mongle 2019). Most hypotheses were tested using phylogenetic ANOVA in *phytools* v0.6.99 (Revell 2012).

**Results.** Phylogenetic signal was virtually identical across the alternative full tree and alternative trees for mammals, birds, lepidosaurs, and amphibians, relative to the primary tree for these groups (Table S2 and S3). Also, rates of evolution in body temperature (Fig. S1) were similar to the primary tree: alternative full tree: 𝜎^2^=0.113 (vs. 0.107 in the primary tree); amphibians: 𝜎^2^=0.175 (vs. 0.165); mammals: 𝜎^2^=0.086 (vs. 0.084); birds: 𝜎^2^=0.078 (vs. 0.078); lepidosaurs: 𝜎^2^=0.127 (vs. 0.115); crocodilians: 𝜎^2^=0.123 (vs. 0.116); and turtles: 𝜎^2^=0.137 (vs. 0.128). Clade-level rates of evolution in ectotherms and endotherms were significantly different across tetrapods (F=10.88, *P*=0.027), and especially after removing amphibians (F=63.16, *P*=0.004), as in the analyses based on the primary tree.

The estimated body temperatures for major ancestral nodes were generally very similar between the primary and alternative trees (almost all within 0.2ºC, except for amphibians; Table S7). Using the alternative tree and evomap (Fig. S2), the body temperature for the ancestor of tetrapods was 28.2ºC (vs. 28.0ºC for the primary tree), for mammals 32.5ºC (vs. 32.3ºC), crocodilians 30.2ºC (vs. 30.1ºC), birds 39.4ºC (vs. 39.4ºC), turtles 27.6ºC (vs. 27.5ºC), lepidosaurs 28.5ºC (vs. 28.5ºC), and amphibians 24.8ºC (vs. 24.0ºC). Note that highest posterior density intervals (HPDI) for each mean value are given in Table S7.

Based on phylogenetic ANOVA of data for individual species in the alternative trees, body temperatures differed significantly among major tetrapod clades (F=1266.48, *P*=0.042; *n*=1709). However, the difference between ectotherms and endotherms was not significant (F=1409.83, *P*=0.069). These results were similar to those from the primary tree.

Comparisons of body temperatures between diel activity states using the alternative tree and phylogenetic ANOVA also yielded similar results to those based on the primary tree. As in the main results, we did not find significant differences between body temperatures of nocturnal and diurnal species across tetrapods (F=203.94, *P*=0.323, *n*=1498), nor within amphibians (F=2.13, *P*=0.553, *n*=106) or mammals (F=32.80, *P*=0.080, *n*=462). Again, there were significant differences within birds (F=45.71, *P*=0.005, *n*=407) and lepidosaurs (F=98.79, *P*=0.046, *n*=489). There were significant differences between nocturnal and diurnal species across ectotherms (F=504.29, *P*=0.007; *n*=629) and across endotherms (F=850.28, *P*=0.009; *n*=869). Using all four diel activity states (i.e. diurnal, nocturnal, arrhythmic and crepuscular) yielded similar results using both the primary (Table S5) and alternative trees (Table S6).

**Appendix S4. Alternative methods for ancestral reconstruction.**

**Methods.** Brownian motion (BM) is one of the most commonly used models in phylogenetic comparative methods (Garamszegi 2014). As typically applied, this model assumes that the change in the variance of a trait is proportional to time, and that the evolutionary rate (𝜎) has a single mean and variance across all branches. Ornstein-Uhlenbeck (OU) models have also been widely used (Garamszegi 2014). OU models involve an optimum value (𝜃) towards which traits are pulled by an adapting force (𝛼). However, many biological traits change at different rates throughout the phylogeny and may show multiple optima (Harvey and Purvis 1991; Garamszegi 2014; Smaers et al. 2016).

In this study, we primarily used a method based on a multiple variance BM (mvBM) model, implemented in the R package *evomap* v0.0.0.9000 (Smaers and Mongle 2019). This method assumes a BM model but allows rate variation across the tree, including branch-specific rates of evolution (Smaers et al. 2016; Smaers and Mongle 2017). First, using maximum likelihood, the estimated ancestral value for each node and the rate of evolution for each branch are estimated. The estimated branch-specific rates of evolution are used to rescale the phylogeny. Then, the rescaled tree is used in a Bayesian Markov chain Monte Carlo (MCMC) framework to incorporate uncertainty in the estimated ancestral values and branch-specific rates of trait evolution. Based on simulations, this method accurately estimates ancestral trait values, both when the trait evolves under a single BM rate across the phylogeny and when there are different rates along different branches of the phylogeny (Smaers et al. 2016).

Rate estimates for each species or clade were obtained using the function “mvBM.getRate” in *evomap* (Smaers and Mongle 2019). This function averages the distribution of estimated rates across the MCMC chains to provide a mean value for each clade or species (Smaers and Mongle 2019). Overall, we used 20,000,000 generations, sampling every 64,000 generations, with a burn-in at 15% of the total number of generations. We ran a total of 20 chains and tested for convergence using the R package *coda* v0.19.1 (Plummer et al. 2006) and Tracer v1.7.1 (Rambaut et al. 2018). The starting point of each chain included the original tree with rescaled branch lengths (Smaers et al. 2016; Smaers and Mongle 2017). The rescaled tree was obtained using the “mvBM” in *evomap* (Smaers and Mongle 2019). All other priors and settings used default values.

An alternative approach was also used to estimate body-temperature evolution across the primary tree, to evaluate whether it gave estimates similar to those from evomap. First, we used the R package *l1ou* v1.42 (Khabbazian et al. 2016). This method identifies the best-fitting set of rate shifts across the tree (Fig. S3), with rates based on OU models. We used the phylogenetic Bayesian Information Criterion (pBIC) for model selection (Khabbazian et al. 2016) and a random root model in the function “estimate_shift_configuration”. We systematically tested a range of values for the maximum number of shifts (5, 10, 15, 20, 25 and 30; Table S8). Models were considered significantly better if the pBIC difference between models was >2 (Burnham and Anderson 2002).

We also estimated the statistical support for each rate shift using this approach. The bootstrap support for each identified shift was estimated using 100 replicates (Table S9). Based on the estimated set of rate shifts, phylogenetically uncorrelated standardized residuals for each node were resampled with replacement to generate bootstrap replicates using the function “l1ou_bootstrap_support” in *l1ou*. Each replicate was analyzed using the *l1ou* method. Bootstrap support for a given shift on a given node was the proportion of bootstrap replicates in which a shift was detected on that node. Shifts were not considered to be supported if bootstrap support was lower than 80%. Therefore, the best-fitting model was identified based on a combination of both model fit and the number of well-supported rate shifts (based on bootstrapping). We note that the cutoff for bootstrap support (80%) was somewhat arbitrary, and other reasonable values could have been used instead (e.g. 95%). However, we found that most inferred shifts in the best-fitting model(s) were well supported using this criterion. Therefore, if use of a different bootstrap cutoff led to supporting a very different number of rate shifts, the results would be very different from the best-fit configuration of rate shifts, which seems problematic.

In addition to identifying the best-fitting configuration of rate shifts, the fit of four *a priori* hypotheses were compared to the fit of the best-fitting set using *l1ou* (Table S8). These four hypotheses were based on the higher mean body temperatures observed in mammals, birds, and lepidosaurs, and the lower mean body temperatures in amphibians. The hypotheses included: (H0) one rate regime across the tree (i.e. single set of parameters with no shifts); (H1) three rate regimes with two shifts, one in extant reptiles (i.e. lepidosaurs, turtles, crocodilians, birds) and another in mammals; (H2) four rate regimes, with shifts in mammals, birds, and lepidosaurs; and (H3) five rate regimes, with shifts in mammals, birds, lepidosaurs, and amphibians.

We also tested whether the best-fitting set of rate shifts and the four hypotheses listed above had better fit to an overall BM or OU model (Table S10). To do this, we used the R package *mvMORPH* v1.1.0 (Clavel et al. 2015). To compare hypotheses, we used the Akaike Information Criterion (AIC), AIC weights, and likelihood-ratio tests. The best-fitting model (model.10 under BM) was then used to estimate ancestral values throughout the tree (Fig. S4). Reconstructions were performed using the function “estim.”

Finally, due to the smaller sample size in amphibians, we tested whether removing this clade substantially impacted the results (Table S11). This involved running the analysis on the tree without amphibians (leaving *n*=1604 species).

**Results.** Using *l1ou,* the best-fitting set of shift configurations was a model with 11 shifts (hereafter, model.11; Table S8, Fig. S3). However, the pBIC difference between a model with 11 or 10 shifts was basically 2 (ΔpBIC=1.98; Table S8). The difference between the two models was a shift identified for the species *Charina bottae* for model.11 (Table S9). We used model.11, since this is the best-fitting model and because the shift in this one species should have little impact on the results regardless. Under model.11, only 10 shifts had bootstrap support ≥80%. Overall, we considered there to be 10 well-supported shifts (bootstrap≥80%; Table S9). We fixed the model to these same shifts (hereafter, model.10). Alternative analyses were based on model.10. Additionally, we applied model.10 with the BM model instead of OU, given the higher support for BM using *mvMORPH* (Table S10).

The ancestral reconstructions based on model.10 with *mvMORPH* were generally similar to the main results (based on *evomap*; Table S7). All were within 2ºC and most were within ~1ºC. We compared these estimates for the major tetrapod clades here. Using *mvMORPH* (Fig. S4), the estimated body temperature for the ancestor of tetrapods was 27.3ºC (versus 28.0ºC using *evomap*). Estimates were also similar between methods for the ancestors of crocodilians (*θ*=28.8ºC vs. 30.1ºC), lepidosaurs (*θ*=27.3ºC vs. 28.5ºC), and turtles (*θ*=26.2ºC vs. 27.5ºC). Estimated values were also similar for the ancestors of mammals (*θ*=34.4ºC vs. 32.3ºC using *evomap*), birds (*θ*=40.3ºC vs. 39.4ºC) and amphibians (*θ*=23.7ºC vs. 24.0ºC). The estimates were also similar after removing amphibians from the analyses (Table S11).

Overall reconstructions were similar across methods using the primary tree. Furthermore, reconstructions were also similar across alternative trees using the same method (*evomap*, see above). Therefore, we did not apply the alternative approach on the alternative tree, given that this would presumably yield results that were very similar to those from the primary method (*evomap*) on the primary tree.

**Appendix S5. Taxon sampling analyses.**

**Methods.** We addressed how incomplete taxon sampling could impact our main conclusions. To do this, we randomly sampled only 10% of the species (*n*=172) from the primary tree. We did this for a total of 10 replicates, each with a different random selection of species. However, the sampling in our main analyses was not entirely random, since all major clades were represented. Therefore, we placed this same restriction on our sampling (i.e. that major clades must be represented even if they might be unsampled if sampling was entirely random). Specifically, we made sure that the following clades were included, with the numbers of species in each clade generally representing 10% of those sampled in the main analyses. Within amphibians, we sampled both salamanders (*n*=8) and frogs (*n*=3). Within lepidosaurs, we sampled the tuatara (*n*=1), snakes (*n*=3), gekkotans (*n*=3), and other lizards (*n*=44). Within mammals, we sampled monotremes (*n*=1); marsupials (*n*=7), and placentals (*n*=49). Within birds, we sampled both paleognaths (*n*=2) and neognaths (*n*=47). Among major clades, we ensured that turtles (*n*=3) and crocodilians (*n*=2) were sampled. We used the function “drop.tip” in *ape* v5.4.1 (Paradis and Schliep 2019) to prune the full tree. The 10 replicate trees are in Dataset S9.

The comparative methods used on each resampled tree were identical to those used in the main analyses. For example, phylogenetic signal was again tested using the function “fitContinuous” in *geiger* v2.0.6.2 (Harmon et al. 2008; Pennell et al. 2014). Ancestral reconstructions were performed using *evomap* v0.0.0.9000 (Smaers and Mongle 2019). Most hypotheses were tested using phylogenetic ANOVA in *phytools* v0.6.99 (Revell 2012). For each set of analyses, we compare our primary results to the average results from across the 10 replicates. For the phylogenetic ANOVA, we summarize the percentage of significant versus non-significant results among replicates.

**Results.** Phylogenetic signal was virtually identical across the resampled trees, relative to the primary tree for these groups with the exception of turtles (Table S2 and Dataset S10): mean across resampled full trees: λ=0.988 (vs. 0.981 in the primary tree); amphibians: λ=0.635 (vs. 0.744); mammals: λ=0.878 (vs. 0.849); birds: λ=0.845 (vs. 0.894); lepidosaurs: λ=0.938 (vs. 0.948); and turtles: 𝜎^2^=0.000 (vs. 0.869). Results for turtles were highly impacted by the very small sample size (*n*=3). These results for turtles are consistent with previous analyses (Jezkova and Wiens 2016) that showed that incomplete sampling can bias model selection towards incorrectly inferring a white-noise model (no phylogenetic signal) when the true model is Brownian motion (high phylogenetic signal). Note that we only included two crocodilians in the resampling analyses, so we could not estimate phylogenetic signal for this clade. In summary, these results supported the idea that overall signal was very high across the tree, but was lower in amphibians.

Rates of evolution in body temperature (Dataset S10) were somewhat lower than those in the primary tree. However, the same patterns were found, with the fastest rates in amphibians, the lowest rates in mammals and birds, and intermediate rates in the other groups. Specifically, the mean rates among the 10 resampled trees were: 𝜎^2^=0.081 (vs. 0.107 in the primary tree); amphibians: 𝜎^2^=0.118 (vs. 0.165); mammals: 𝜎^2^=0.055 (vs. 0.084); birds: 𝜎^2^=0.053 (vs. 0.078); lepidosaurs: 𝜎^2^=0.099 (vs. 0.115); crocodilians: 𝜎^2^=0.091 (vs. 0.116); and turtles: 𝜎^2^=0.097 (vs. 0.128). Clade-level rates of evolution were significantly different across tetrapods for 50% of the replicates (Dataset S10), supporting the difference between ectotherms and endotherms. The lower rates were somewhat surprising given previous simulations and subsampling analyses (e.g. Jezkova and Wiens 2016), which showed little or no impact of subsampling on estimated rates. The difference may be explained by the fact that our subsampling here was not fully random (i.e. each replicate included some species from each major clade, as in our main analyses).

The estimated body temperatures for major ancestral nodes were generally very similar between the resampled and primary trees (generally within 0.6ºC; Tables S7 and Dataset S10). Using the mean across resampled full trees, the body temperature for the ancestor of tetrapods was 29.7ºC (vs. 28.0ºC for the primary tree), for mammals 32.9ºC (vs. 32.3ºC), crocodilians 30.2ºC (vs. 30.1ºC), birds 39.4ºC (vs. 39.4ºC), turtles 27.7ºC (vs. 27.5ºC), lepidosaurs 29.9ºC (vs. 28.5ºC), and amphibians 24.2ºC (vs. 24.0ºC). Again, the relative differences between body temperatures at the ancestral nodes were similar to the primary results, with higher ancestral temperatures in birds and mammals, lower temperatures in amphibians, and values for the other clades close to that for the tetrapod ancestor. The highest posterior density intervals (HPDI) for each mean value are given in Dataset S10.

Based on phylogenetic ANOVA of data for individual species in the resampled trees, body temperatures differed significantly among major tetrapod clades for 70% of the trees (Dataset S10). However, the difference between ectotherms and endotherms was significant only for 30% of the trees (Dataset S10). These results were similar to those from the primary tree.

Comparisons of body temperatures between diel activity states using the resampled trees and phylogenetic ANOVA also yielded similar results to those based on the primary tree (Dataset S10). As in the main results, we did not find support for significant differences between body temperatures of nocturnal and diurnal species across tetrapods (0% of replicates), nor within amphibians (0%), turtles (0%) or mammals (10%). Again, there was often support for significant differences within birds (70% of replicates) and lepidosaurs (60%). There was support for significant differences between nocturnal and diurnal species across ectotherms (100%) and across endotherms (80%). Using all four diel activity states (i.e. diurnal, nocturnal, arrhythmic and crepuscular) yielded similar results (Dataset S10).

We note that some subsampling replicates yielded non-significant results for phylogenetic ANOVA. This is consistent with simulations (Ackerly 2000) that showed that incomplete taxon sampling can reduce statistical power for phylogenetic comparative methods (i.e. fewer correct, significant results) but only rarely generates false positives (i.e. significant but incorrect results).

**Appendix S6. Relationships between climate and body temperature in crocodilians and turtles.**

**Methods.** We tested relationships between macroclimatic temperature variables and body temperatures among species of crocodilians and turtles, using phylogenetic regression. We generally followed the same methods used in a recent study that examined these relationships in other major tetrapod groups (Qu and Wiens 2020). We obtained data on three standard climatic measures of temperature: mean annual temperature (Bio1), maximum temperature of the warmest month (Bio5), and minimum temperature of the coldest month (Bio6). These represent the mean and extreme climatic temperatures that each species experiences within its geographic range. Climatic data for crocodilians were obtained from a previous study (Quintero and Wiens 2013).

We estimated the climatic data for turtles in this study. Occurrence records for turtle species were obtained from the distributional database of the Global Biodiversity Information Facility (Chamberlain et al. 2020), which includes a compilation of records from natural history collections. To ensure data quality, we checked if the locality records were consistent with available range maps (IUCN 2020). If these range maps were unavailable, we used descriptions of the geographic distribution of each species (Uetz et al. 2019) to check for any problematic localities (e.g. localities outside the known range, human introductions, occurrence records on land for marine species). These problematic localities were then removed. Overall, we obtained multiple unique locality records for each turtle species (mean=274, range=3–496). Note: the species with 3 localities was an endangered species imperiled by overcollecting, so relatively few known localities have been made publicly available.

Climatic data were obtained from the WorldClim dataset at ~1 km^2^ spatial resolution (Hijmans et al. 2005). We extracted climatic data for each turtle species occurrence record using the R packages *rgdal* v1.4.8 (Bivand et al. 2019) and *raster* v3.0.7 (Hijmans 2020). Then, we obtained a single value for each species for each climatic variable by averaging values across the localities for each species. Data on climate for each species are provided in Dataset S8.

We tested for a relationship between each climatic variable and body temperature using phylogenetic generalized least squares (PGLS) regression (Martins and Hansen 1997). We implemented PGLS using the R package *caper* v1.0.1 (Orme et al. 2018). We generated reduced trees to separately analyze turtles and crocodilians using the function “comparative.data” in *caper*.

**Results.** We found no significant relationship between any of the three climatic variables and body temperatures among the 30 species of turtles with body-temperature data (Bio1: coefficient=0.005, *P*=0.974, *r*^2^<0.001; Bio5: coefficient=0.035, *P*=0.877, *r*^2^=0.001; Bio6: coefficient=-0.002, *P*=0.981, *r*^2^<0.001). We also found no significant relationship among the 11 crocodilians with body-temperature data (Bio1: coefficient=-0.502, *P*=0.153, *r*^2^=0.213; Bio5: coefficient=-0.127, *P*=0.751, *r*^2^=0.012; Bio6: coefficient=-0.317, *P*=0.108, *r*^2^=0.261).

**Appendix References**

Ackerly, D. D. 2000. Taxon sampling, correlated evolution, and independent contrasts. Evolution 54:1480–1492.

Anderson, S. R., and J. J. Wiens. 2017. Out of the dark: 350 million years of conservatism and evolution in diel activity patterns in vertebrates. Evolution 71:1944–1959.

Aschoff, V. J. 1981. Der tagesgang der körpertemperatur und der sauerstoffaufnahme bei slugetieren als funktion des Körpergewichtes. Z. Säugetierkd. 46:201–216.

Bivand, R., T. Keitt, and B. Rowlingson. 2019. rgdal: bindings for the “Geospatial” data abstraction library. R package version 1.4-8. https://CRAN.R-project.org/package=rgdal.

Burnham, K. P., and D. R. Anderson. 2002. Model selection and multimodel inference: a practical information-theoretic approach. Springer, New York.

Chamberlain, S., V. Barve, D. Mcglinn, D. Oldoni, P. Desmet, L. Geffert, et al. 2020. rgbif: interface to the Global Biodiversity Information Facility API_. R package version 3.2.0, <URL: https://CRAN.R-project.org/package=rgbif>.

Clarke, A., and P. Rothery. 2008. Scaling of body temperature in mammals and birds. Funct. Ecol. 22:58–67.

Clarke, A., P. Rothery, and N. J. B. Isaac. 2010. Scaling of basal metabolic rate with body mass and temperature in mammals. J. Anim. Ecol. 79:610–619.

Clavel, J., G. Escarguel, and G. Merceron. 2015. mvMORPH: an R package for fitting multivariate evolutionary models to morphometric data. Methods Ecol. Evol. 6:1311–1319.

Ericson, P. G. P., C. L. Anderson, T. Britton, A. Elzanowski, U. S. Johansson, M. Källersjö, et al. 2006. Diversification of Neoaves: integration of molecular sequence data and fossils. Biol. Lett. 2:543–547.

Faurby, S., M. Davis, R. Ø. Pedersen, S. D. Schowanek, A. Antonelli1, and J. Svenning. 2018. PHYLACINE 1.2: the phylogenetic atlas of mammal macroecology. Ecology 99:2626–2626.

Garamszegi, L. Z. 2014. Modern phylogenetic comparative methods and their application in evolutionary biology: concepts and practice. Springer, Berlin.

Garland, T., A. W. Dickerman, C. M. Janis, and J. A. Jones. 1993. Phylogenetic analysis of covariance by computer simulation. Syst. Biol. 42:265–292.

Glazier, D. S. 2009. Activity affects intraspecific body-size scaling of metabolic rate in ectothermic animals. J. Comp. Physiol. B 179:821–828.

Hackett, S. J., R. T. Kimball, S. Reddy, R. C. K. Bowie, E. L. Braun, M. J. Braun, et al. 2008. A phylogenomic study of birds reveals their evolutionary history. Science 320:1763–1768.

Harmon, L. J., J. T. Weir, C. D. Brock, R. E. Glor, and W. Challenger. 2008. GEIGER: investigating evolutionary radiations. Bioinformatics 24:129–131.

Harvey, P. H., and A. Purvis. 1991. Comparative methods for explaining adaptations. Nature 351:619–624.

Hijmans, R. J. 2020. raster: geographic data analysis and modeling. R package version 3.4-5. http://CRAN.R-project.org/package=raster.

Hijmans, R. J., S. E. Cameron, J. L. Parra, P. G. Jones, and A. Jarvis. 2005. Very high resolution interpolated climate surfaces for global land areas. Int. J. Climatol. 25:1965–1978.

Hill, R. W., G. A. Wyse, and M. Anderson. 2016. Animal physiology. Sinauer, Massachusetts.

IUCN. 2020. The IUCN Red List of Threatened Species. Version 2020-2. https://www.iucnredlist.org. Downloaded on 09 July 2020.

Jetz, W., and R. A. Pyron. 2018. The interplay of past diversification and evolutionary isolation with present imperilment across the amphibian tree of life. Nat. Ecol. Evol. 2:850–858.

Jetz, W., G. H. Thomas, J. B. Joy, K. Hartmann, and A. O. Mooers. 2012. The global diversity of birds in space and time. Nature 491:444–448.

Jezkova, T., and J. J. Wiens. 2016. Rates of change in climatic niches in plant and animal populations are much slower than projected climate change. Proc. R. Soc. B Biol. Sci. 283:20162104.

Khabbazian, M., R. Kriebel, K. Rohe, and C. Ané. 2016. Fast and accurate detection of evolutionary shifts in Ornstein-Uhlenbeck models. Methods Ecol. Evol. 7:811–824.

Martins, E. P., and T. F. Hansen. 1997. Phylogenies and the comparative method: a general approach to incorporating phylogenetic information into the analysis of interspecific data. Am. Nat. 149:646–667.

Meredith, R. W., J. E. Janecka, J. Gatesy, O. A. Ryder, C. A. Fisher, E. C. Teeling, et al. 2011. Impacts of the Cretaceous terrestrial revolution and KPg extinction on mammal diversification. Science 334:521–524.

Orme, D., R. Freckleton, G. Thomas, T. Petzoldt, S. Fritz, N. Isaac, et al. 2018. caper: comparative analyses of phylogenetics and evolution in R. R package version 1.0.1. https://CRAN.R-project.org/package=caper.

Paradis, E., and K. Schliep. 2019. ape 5.4-1: an environment for modern phylogenetics and evolutionary analyses in R. Bioinformatics 35:526–528.

Pennell, M. W., J. M. Eastman, G. J. Slater, J. W. Brown, J. C. Uyeda, R. G. FitzJohn, et al. 2014. geiger v2.0: an expanded suite of methods for fitting macroevolutionary models to phylogenetic trees. Bioinformatics 30:2216–2218.

Plummer, M., N. Best, K. Cowles, and K. Vines. 2006. CODA: convergence diagnosis and output analysis for MCMC. R News 6:7–11.

Prinzinger, R., A. Preßmar, and E. Schleucher. 1991. Body temperature in birds. Comp. Biochem. Physiol. Part A Physiol. 99:499–506.

Pyron, R. A., and J. J. Wiens. 2013. Large-scale phylogenetic analyses reveal the causes of high tropical amphibian diversity. Proc. R. Soc. B Biol. Sci. 280:20131622.

Qu, Y.-F., and J. J. Wiens. 2020. Higher temperatures lower rates of physiological and niche evolution. Proc. R. Soc. B Biol. Sci. 287:20200823.

Quintero, I., and J. J. Wiens. 2013. Rates of projected climate change dramatically exceed past rates of climatic niche evolution among vertebrate species. Ecol. Lett. 16:1095–1103.

Rambaut, A., A. J. Drummond, D. Xie, G. Baele, and M. A. Suchard. 2018. Posterior summarization in Bayesian phylogenetics using Tracer 1.7. Syst. Biol. 67:901–904.

Revell, L. J. 2012. phytools: an R package for phylogenetic comparative biology (and other things). Methods Ecol. Evol. 3:217–223.

Rolland, J., F. L. Condamine, F. Jiguet, and H. Morlon. 2014. Faster speciation and reduced extinction in the tropics contribute to the mammalian latitudinal diversity gradient. PLoS Biol. 12:e1001775.

Seebacher, F. 2009. Responses to temperature variation: integration of thermoregulation and metabolism in vertebrates. J. Exp. Biol. 212:2885–2891.

Smaers, J. B., and C. S. Mongle. 2019. evomap: Evomap. R package version 0.0.0.9000.

Smaers, J. B., and C. S. Mongle. 2017. On the accuracy and theoretical underpinnings of the multiple variance Brownian motion approach for estimating variable rates and inferring ancestral states. Biol. J. Linn. Soc. 121:229–238.

Smaers, J. B., C. S. Mongle, and A. Kandler. 2016. A multiple variance Brownian motion framework for estimating variable rates and inferring ancestral states. Biol. J. Linn. Soc. 118:78–94.

Tattersall, G. J. 2016. Reptile thermogenesis and the origins of endothermy. Zoology 119:403–405. Elsevier GmbH.

Tattersall, G. J., C. A. C. Leite, C. E. Sanders, V. Cadena, D. V. Andrade, A. S. Abe, et al. 2016. Seasonal reproductive endothermy in tegu lizards. Sci. Adv. 2:e1500951.

Tonini, J. F. R., K. H. Beard, R. B. Ferreira, W. Jetz, and R. A. Pyron. 2016. Fully-sampled phylogenies of squamates reveal evolutionary patterns in threat status. Biol. Conserv. 204:23–31.

Uetz, P., P. Freed, and J. Hošek. 2019. The Reptile Database. <http://www.reptile-database.org>. Accessed 12 August 2019.

Upham, N. S., J. A. Esselstyn, and W. Jetz. 2019. Inferring the mammal tree: species-level sets of phylogenies for questions in ecology, evolution, and conservation. PLoS Biol. 17:e3000494.

Zheng, Y., and J. J. Wiens. 2016. Combining phylogenomic and supermatrix approaches, and a time-calibrated phylogeny for squamate reptiles (lizards and snakes) based on 52 genes and 4162 species. Mol. Phylogenet. Evol. 94:537–547.

**Figure S1.** Estimated rates of body-temperature evolution within each major clade using the alternative tree. The distribution of estimated rates (density) for each major clade for body temperature evolution (𝜎^2^) is shown. The alternative tree includes 1709 species. The distribution of estimated rates is based on all internal and terminal branches for each clade.

**Figure S2.** Reconstructed evolution of body temperatures using *evomap* on the alternative tree. The alternative tree includes 1709 species. Silhouettes courtesy of PhyloPic: T. Michael Keesey (mammal; Public Domain Dedication 1.0 license), B. Kimmel (crocodilian; Public Domain Dedication 1.0 license), George Edward Lodge (bird; Public Domain Dedication 1.0 license), Scott Hartman (turtle; Creative Commons Attributions 3.0 Unported license), Michael Scroggie (lepidosaur; Public Domain Dedication 1.0 license) and Steven Traver (amphibian; Public Domain Dedication 1.0 license).

**Figure S3.** Estimated rate shifts in body temperature across tetrapods using *11ou*. Results are based on the primary tree (*n*=1721 species). The best-fitting OU model included 11 shifts (hereafter, model.11; Table S8). Each asterisk is an identified rate shift. Shifts are associated with large-scale named clades (1: mammals; 2: birds; 4: the lizard clade Iguania; 6: the lizard family Lacertidae; 7: the lizard family Teiidae; 9: amphibians; 10: salamanders, excluding *Pseudobranchus striatus*), smaller clades within families and genera (11: the tropical salamander genus *Bolitoglossa* (4 species)), and individual species (3: the temperate boa, *Charina bottae*; 5: the lizard, *Lophosaurus spinipes*; 8: the tuatara, *Sphenodon punctatus*).

Figure S4. Ancestral reconstructions of body temperature using *mvMorph*. Results shown are based on model.10 (based on the BM model) and the primary tree (*n*=1721 species). Model.10 includes only shifts with bootstrap support >80%. The well-supported shifts are: (1) mammals (*θ*=34.4ºC); (2) birds (*θ*=40.3ºC); (3) the snake *Charina bottae* (*θ*=14.8ºC); (4) the lizard clade Iguania (*θ*=33.7ºC); (5) the lizard species *Lophosaurus spinipes* (*θ*=19ºC); (6) the lizard family Teiidae (*θ*=36.6ºC); (7) the tuatara, *Sphenodon punctatus* (*θ*=14.5ºC); (8) amphibians (*θ*=23.7ºC); (9) salamanders (minus *Pseudobranchus striatus*; *θ*=13.6ºC); and (10) 4 *Bolitoglossa* species (*θ*=22.6ºC). The estimate for the root was 27.3ºC. Silhouettes courtesy of PhyloPic: T. Michael Keesey (mammal; Public Domain Dedication 1.0 license), George Edward Lodge (bird; Public Domain Dedication 1.0 license), V. Deepak (snake; Creative Commons Attributions 3.0 Unported license), Sarah Werning (*Anolis*; Creative Commons Attributions 3.0 Unported license), Michael Scroggie (Amphibolurinae; Public Domain Dedication 1.0 license), Kailah Thorn and Ben King (*Egernia saxatilis*; Public Domain Dedication 1.0 license), Steven Traver (*Sphenodon punctatus* and *Bufo*; Public Domain Dedication 1.0 license), and C. Camilo Julián-Caballero (salamander; Creative Commons Attributions 3.0 Unported license).

**Table S1.** Percentage of sampled species with each diel activity state in each clade and category.

| Clades | Species  sampled | Diurnal (%) | Nocturnal (%) | Arrhythmic (%) | Crepuscular (%) |
| --- | --- | --- | --- | --- | --- |
| Mammals | 571 | 19.61 | 62.52 | 14.19 | 3.68 |
| Crocodilians | 11 | 0 | 100.00 | 0 | 0 |
| Birds | 474 | 81.01 | 4.85 | 11.18 | 2.95 |
| Turtles | 30 | 66.67 | 10.00 | 16.67 | 6.67 |
| Lepidosaurs | 518 | 82.05 | 12.36 | 4.44 | 1.16 |
| Amphibians | 117 | 6.84 | 83.76 | 8.55 | 0.85 |
| Ectotherms | 676 | 67.01 | 26.04 | 5.62 | 1.33 |
| Endotherms | 1045 | 47.46 | 36.36 | 12.82 | 3.35 |

**Table S2.** Estimated phylogenetic signal and evolutionary rates for body temperature in tetrapods for the primary tree. Phylogenetic signal is estimated using Pagel’s (1999) λ. Note that the λ value for crocodilians is not reliable: a preliminary analysis with one additional species (*n*=12) had λ=0.941. Furthermore, simulations and resampling analyses suggest that phylogenetic signal may be systematically underestimated when the number of taxa is this small, whereas the estimated rates appear to be minimally affected (Jezkova and Wiens 2016). The mean rate (𝜎^2^ clade level) is averaged across the distribution of estimated rates, considering all internal and terminal branches for the full tree and each major clade. The species-level rate (𝜎^2^ species-level) indicates the mean of the species-level rates within clades. Within mammals, we found three pairs of species with exceptionally fast rates (>1; in the genera *Meriones*, *Rousettus*, and *Rhinolophus*; Dataset S7). Excluding these 6 species (out of 571) has a dramatic impact on the mean rate for mammals (value in parentheses). Given the sensitivity of the species-level results to extreme values for individual species, we do not base our conclusions on the species-level analyses.

| Clade | λ | 𝜎^2^  (clade-level) | 𝜎^2^  (species-level) |
| --- | --- | --- | --- |
| All species (*n*=1721) | 0.981 | 0.107 | 0.130 |
| Mammals (*n*=571) | 0.849 | 0.084 | 0.160 (0.109) |
| Crocodilians (*n*=11) | 0.000 | 0.116 | 0.141 |
| Birds (*n*=474) | 0.894 | 0.078 | 0.084 |
| Turtles (*n*=30) | 0.869 | 0.128 | 0.140 |
| Lepidosaurs (*n*=518) | 0.948 | 0.115 | 0.128 |
| Amphibians (*n*=117) | 0.744 | 0.165 | 0.179 |

**Table S3.** Estimated phylogenetic signal and evolutionary rates for body temperature in tetrapods for the alternative tree. Phylogenetic signal was estimated using Pagel’s λ. Signal was estimated across the full tree and the alternative trees for mammals, birds, lepidosaurs, and amphibians. The mean rate (𝜎^2^) is averaged across the distribution of estimated rates, considering all internal and terminal branches for the full tree and each major clade. Although we did not use alternative trees within crocodilians and turtles, we still obtained different mean clade-level rates within these two groups. Rates within these two groups are estimated incorporating the full tree, and so their rate estimates were impacted (slightly) by the different topologies across the tree. Note that the estimate of λ in crocodilians was not considered reliable given the small sample size (see Table S2).

| Clade | λ | 𝜎^2^  (clade-level) |
| --- | --- | --- |
| Alternative all species (*n*=1709) | 0.979 | 0.113 |
| Alternative mammals (*n*=559) | 0.876 | 0.086 |
| Alternative crocodilians (*n*=11) | 0.000 | 0.123 |
| Alternative birds (*n*=474) | 0.891 | 0.078 |
| Alternative turtles (*n*=30) | 0.869 | 0.137 |
| Alternative lepidosaurs (*n*=518) | 0.948 | 0.127 |
| Alternative amphibians (*n*=117) | 0.705 | 0.175 |

**Table S4.** Results of phylogenetic ANOVA testing for differences in body temperatures across major tetrapod clades, using the primary tree. Results are based on post-hoc tests. Significant values (*P*<0.05) are boldfaced. t: t-test statistic; *P*: *P*-value of the t-test.

| Clade | Crocodilians | | Birds | | | | | Turtles | | Lepidosaurs | | | | Amphibians | | |
| --- | --- | --- | --- | --- | --- | --- | --- | --- | --- | --- | --- | --- | --- | --- | --- | --- |
|  | t | *P* | | t | | *P* | | t | *P* | t | *P* | | t | | | *P* |
| Mammals | 6.88 | 1.000 | | | -24.65 | | 1.000 | 16.14 | 0.959 | 23.04 | 1.000 | **59.80** | | | **0.029** | |
| Crocodilians | - | - | | | - | | - | - | - | - | - | - | | | - | |
| Birds | 11.89 | 0.475 | | | - | | - | - | - | - | - | - | | | - | |
| Turtles | -2.64 | 1.000 | | | -24.20 | | 0.139 | - | - | - | - | - | | | - | |
| Lepidosaurs | 2.28 | 1.000 | | | -46.09 | | 0.610 | 8.66 | 1.000 | - | - | - | | | - | |
| Amphibians | -12.60 | 0.475 | | | **-73.62** | | **0.008** | -14.88 | 0.959 | -45.63 | 0.107 | - | | | - | |

**Table S5.** Results of phylogenetic ANOVA testing for a relationship between diel activity and body temperatures, using all four diel-activity states on the primary tree. Significant values (*P*<0.05) are boldfaced. F: F-statistic; *P*: *P*-value of the F-statistic.

| Clade | Diel | |
| --- | --- | --- |
|  | F | *P* |
| All species (*n*=1721) | 69.37 | 0.338 |
| Mammals (*n*=571) | 13.43 | 0.100 |
| Birds (*n*=474) | **17.69** | **0.008** |
| Turtles (*n*=30) | 2.98 | 0.294 |
| Lepidosaurs (*n*=518) | **41.61** | **0.029** |
| Amphibians (*n*=117) | 2.20 | 0.434 |
| Ectotherms (*n*=676) | **170.54** | **0.002** |
| Endotherms (*n*=1045) | **251.59** | **0.008** |

**Table S6.** Results of phylogenetic ANOVA testing for a relationship between diel activity and body temperatures, using all four diel activity states and the alternative tree. Significant values (*P*<0.05) are boldfaced. F: F-statistic; *P*: *P*-value of the F-statistic.

| Clade | Diel | |
| --- | --- | --- |
|  | F | *P* |
| Alternative all species (*n*=1709) | 69.00 | 0.347 |
| Alternative mammals (*n*=559) | 11.50 | 0.152 |
| Alternative birds (*n*=474) | **17.69** | **0.012** |
| Alternative lepidosaurs (*n*=518) | **41.61** | **0.028** |
| Alternative amphibians (*n*=117) | 2.20 | 0.493 |
| Alternative ectotherms (*n*=676) | **170.54** | **0.006** |
| Alternative endotherms (*n*=1033) | **249.44** | **0.009** |

**Table S7.** Estimated ancestral body temperatures (Tb ºC) and the 95% highest posterior density interval (HPDI) for the ancestral node of each major clade. Results are summarized for the primary tree and the alternative tree**.**

| Clade | Primary tree | | Alternative tree | |
| --- | --- | --- | --- | --- |
|  | Tb ºC [HPDI] | *n* | Tb ºC [HPDI] | *n* |
| All species | 28.0 [23.7–32.4] | 1721 | 28.2 [24.0–32.5] | 1709 |
| Mammals | 32.3 [28.8–35.6] | 571 | 32.5 [29.2–35.9] | 559 |
| Crocodilians | 30.1 [27.3–32.9] | 11 | 30.2 [27.2–32.9] | 11 |
| Birds | 39.4 [37.5–41.4] | 474 | 39.4 [37.5–41.4] | 474 |
| Turtles | 27.5 [23.6–31.3] | 30 | 27.6 [23.7–31.5] | 30 |
| Lepidosaurs | 28.5 [24.0–32.9] | 518 | 28.5 [23.6–33.4] | 518 |
| Amphibians | 24.0 [20.2–27.9] | 117 | 24.8 [20.8–29.1] | 117 |

**Table S8.** Results of Ornstein-Uhlenbeck model testing. The 11ou approach was used to compare the support for different estimated numbers of shifts (after changing the maximum number of shifts allowed) and for four *a priori* hypotheses. The best-fitting models had 11 shifts (hereafter, model.11). Models include the phylogenetic placement, direction, and magnitude of shifts in the evolution of body temperature. max.nShifts: the maximum number of shifts the function is allowed to estimate; nShifts: the actual number of estimated shifts; pBIC: phylogenetic Bayesian Information Criterion (BIC); ΔpBIC: the difference between the values of pBIC from each model and the best-fitting model; H0: one rate regime across all tree; H1: three rates regime with two shifts (i.e. reptiles and mammals); H2: four rates regime with three shifts (mammals, birds, and lepidosaurs); H3: five rates regime with four shifts (mammals, birds, lepidosaurs, and amphibians). For hypothesis testing, no maximum number of rate shifts was set (hence we list these as “NA”).

| Tree | max.nShifts | nShifts | pBIC | ΔpBIC |
| --- | --- | --- | --- | --- |
| All species (*n*=1721) | 5 | 3 | 7933.19 | 45.13 |
|  | 10 | 4 | 7927.06 | 39.00 |
|  | 15 | 10 | 7890.04 | 1.98 |
|  | 20 | 10 | 7890.04 | 1.98 |
|  | 25 | 11 | 7888.06 | - |
|  | 30 | 11 | 7888.06 | - |
| H0: single rate regime | NA | 0 | 7975.37 | 87.31 |
| H1: three rates regime | NA | 2 | 7966.75 | 78.69 |
| H2: four rates regime | NA | 3 | 7970.44 | 82.38 |
| H3: five rates regime | NA | 4 | 7964.63 | 76.57 |

**Table S9.** Location of rate shifts and support for each shift for the two best-fitting OU models. Results are based on OU models implemented using the 11ou approach (model comparisons in Table S8) and using the primary tree. Bootstrap support values are based on 100 iterations. Shifts with bootstrap support values >80% are boldfaced.

| Node | model.11 | |
| --- | --- | --- |
|  | Shift values | Bootstrap |
| 1) mammals | **7.38** | **94%** |
| 2) birds | **12.28** | **99%** |
| 3) *Charina bottae* (snake) | **-15.57** | **80%** |
| 4) Iguania (lizard clade) | **5.36** | **92%** |
| 5) *Lophosaurus spinipes* (lizard) | **-29.03** | **98%** |
| 6) Lacertidae (lizard family) | 6.85 | 41% |
| 7) Teiidae (lizard family) | **9.77** | **83%** |
| 8) *Sphenodon punctatus* (tuatara) | **-13.81** | **86%** |
| 9) amphibians | **-4.93** | **93%** |
| 10) salamanders (minus *Pseudobranchus striatus*) | **-9.74** | **88%** |
| 11) 4 *Bolitoglossa* species (salamanders) | **13.95** | **85%** |

**Table S10.** Relative support for different models of body-temperature evolution. Models included multiple rates of evolution under a Brownian motion (BM) or Ornstein-Uhlenbeck (OU) model of evolution. We compared four *a priori* hypotheses and model.10 from the *l1ou* analyses (Appendix S2). Analyses were done using *mvMORPH*, and used the primary tree. Model.10 under BM is the best-fitting model (boldfaced). H0: one rate regime across all tree; H1: three rates regime with two shifts (i.e. reptiles and mammals); H2: four rates regime with three shifts (i.e. mammals, birds, and lepidosaurs); H3: five rates regime with four shifts (i.e. mammals, birds, lepidosaurs, and amphibians); model.10: model including the 10 shifts with bootstrap support above/equal to 80%; AIC: Akaike Information Criterion.

| Evolutionary model | Model | AIC | AIC weights |
| --- | --- | --- | --- |
| BM | H0: single rate regime | 8033.86 | <0.001 |
|  | H1: three rates regime | 7964.32 | <0.001 |
|  | H2: four rates regime | 7741.55 | <0.001 |
|  | H3: five rates regime | 7743.65 | <0.001 |
|  | **model.10** | **7707.54** | **~0.91** |
| OU | H0: single rate regime | 7962.83 | <0.001 |
|  | H1: three rates regime | 7918.71 | <0.001 |
|  | H2: four rates regime | 7904.69 | <0.001 |
|  | H3: five rates regime | 7879.46 | <0.001 |
|  | model.10 | 7712.09 | ~0.09 |

**Table S11.** Model comparisons for the evolution of body temperature (using 11ou) after excluding amphibians. The number of rate shifts was estimated after setting different values for the maximum number of shifts (max.nShifts). Setting max.nShifts to 20, 25 or 30 yielded the same results (10 shifts in body temperature). These ten shifts occurred in the following clades and species: (1) reptiles (including birds; bootstrap support=96%); (2) birds (100%); (3) *Charina bottae* (90%); (4) Iguania (96%); (5) *Lophosaurus spinipes* (98%); (6) Lacertidae (69%); (7) Teiidae (89%); (8) Scincidae (a clade including 32 out of 85 sampled species of Scincidae; 63%); (9) Scincidae (a clade including 24 out of 85 sampled species of Scincidae; 64%); and (10) *Sphenodon punctatus* (93%). Only 7 shifts had bootstrap support >80%. Using these 7 shifts, the best fitting model is BM (hereafter, model.7.noamphibian: AIC_BM_=7035.91 and AIC_OU_=7059.08). Under model.7.noamphibian, the point estimate for the ancestral node of mammals is 34.4ºC (versus 34.4ºC when we included amphibians in model.10). Estimated ancestral values for other clades under model.7.noamphibian were also similar to model.10, including crocodilians (*θ*=28.8ºC vs. 28.8ºC in model.10), lepidosaurs (*θ*=27.3ºC vs. 27.3ºC), turtles (*θ*=26.2ºC vs. 26.2ºC) and birds (*θ*=40.3ºC vs. 40.3ºC). nShifts: number of estimated shifts; pBIC: phylogenetic Bayesian Information Criterion (BIC); ΔpBIC: reflects the difference between the values of pBIC from each model and the best-fitting model.

| Tree | max.nShifts | nShifts | pBIC | ΔpBIC |
| --- | --- | --- | --- | --- |
| Without amphibians (*n*=1604) | 5 | 3 | 7226.24 | 59.29 |
|  | 10 | 5 | 7198.25 | 31.30 |
|  | 15 | 6 | 7193.24 | 26.29 |
|  | 20 | 10 | 7166.95 | 0 |
|  | 25 | 10 | 7166.95 | 0 |
|  | 30 | 10 | 7166.95 | 0 |
